# Digital Compensation? Investigating the Moderating Role of the Home Learning Environment in the Effectiveness of Learning Apps

**DOI:** 10.3390/bs16081304

**Published:** 2026-07-31

**Authors:** Tina Schiele, María Valcárcel Jiménez, Öykü Camligüney, Astrid Wirth, Frank Niklas

**Affiliations:** 1 Department of Psychology, University of Munich, Leopoldstr. 13, 80802 Munich, Germany; maria.valcarcel@psy.lmu.de (M.V.J.); oeykue.camligueney@psy.lmu.de (Ö.C.); niklas@psy.lmu.de (F.N.); 2 Department of Education, University of Vienna, Universitätsring 1, 1010 Vienna, Austria; astrid.wirth@univie.ac.at

**Keywords:** learning apps, early literacy, early numeracy, home literacy environment, home numeracy environment, Learning4Kids

## Abstract

Children already differ in their literacy and numeracy skills before school entry, which partly is due to differences in their home literacy and numeracy environments (HLE/HNE). Learning apps may serve as a compensatory resource when parents cannot provide adequate support. Based on this assumption, this study investigates whether learning apps predict gains in literacy and numeracy skills, and whether HLE/HNE quality moderates this relation. *N* = 500 children from Germany (*M*_age_ = 60.69 months, *SD* = 4.61) received either literacy, numeracy, or no learning apps for a period of six months before and after which literacy and numeracy skills were assessed. The HLE/HNE were measured via parent questionnaires at T1. Regression models showed that being in the numeracy app intervention group was associated with greater numeracy gains compared to a business-as-usual control group, while the literacy app intervention group showed greater literacy gains compared to all other groups. However, HLE/HNE did not emerge as significant moderators, providing limited evidence for digital compensation of lower-quality HLEs/HNEs. Methodological considerations that call for caution in interpreting the findings are discussed. The findings evaluate the promising character of learning apps to supplement learning in analog HLE/HNE. The potential impact of quality vs. quantity of app usage for compensatory effects is discussed.

## 1. Introduction

International comparative studies reveal substantial educational shortcomings in the literacy and numeracy performances of young children in Germany and show marked declines particularly for literacy compared to earlier cohorts (McElvany et al., 2023; Schwippert et al., 2024). Such substantial difficulties in reading, writing, and numerical understanding often persist, and performance gaps tend to widen during children’s educational trajectories (Niklas et al., 2015). However, children do not enter school as blank slates encountering literacy and numeracy for the first time on their first day of school. Rather, the foundations of their academic development are laid well before school entry.

A key context in which these foundational skills develop is the home learning environment. It plays a crucial role in the cognitive development of young children by providing learning resources, parental support, and cognitively stimulating activities that foster early literacy and numeracy skills (Bradley & Corwyn, 2002; Bronfenbrenner, 1979; Melhuish et al., 2008). However, the quality of both the home literacy environment (HLE) and the home numeracy environment (HNE) varies depending on child (e.g., age, sex, intelligence) and family (e.g., socioeconomic status [SES], migration background) characteristics (Mues et al., 2024; Valcárcel Jiménez et al., 2025).

Digital learning opportunities may help address the disparities in the quality of the HLE and HNE, thus reducing educational gaps. Most families with preschool-aged children in Germany own mobile devices such as tablet computers (i.e., tablets) and smartphones (Medienpädagogischer Forschungsverbund Südwest (mpfs), 2024). Further, learning apps provide a relatively low-cost way to access educational experiences (Niklas et al., 2025; Schiele et al., 2025). Studies indicate small to moderate effects of learning apps on early literacy and numeracy skills (meta-analysis: Kim et al., 2021; review: Neumann & Neumann, 2017). However, their effectiveness may depend on the quality of the HLE or HNE. A high-quality HLE typically includes a high frequency of activities such as shared reading or letter learning instruction, whereas a high-quality HNE involves frequent joint activities such as counting or simple arithmetic. Previous research suggests that high-quality digital learning environments can complement and strengthen analog learning activities (Lehrl et al., 2021). Accordingly, learning apps may be particularly beneficial for children who grow up in lower-quality HLEs or HNEs, by compensating for limited analog learning opportunities through their comprehensive availability and flexible, relatively low-cost selection of learning materials.

The present study therefore examines whether engaging with researcher-developed high-quality learning apps for six months can predict immediate gains in literacy and numeracy skills among preschool children. Additionally, it investigates whether the quality of the HLE or HNE moderates the effectiveness of these apps in promoting skill gains, while controlling for relevant child and family characteristics.

### 1.1. Early Literacy and Numeracy Skills

Emergent literacy skills can be distinguished into inside-out and outside-in skills (Whitehurst & Lonigan, 1998). Inside-out skills include phonological awareness and letter knowledge, which are central for decoding words and phonemes. Phonological awareness refers to the ability to detect and manipulate the sound structure of language (Sénéchal et al., 2004), while letter knowledge encompasses children’s familiarity with the alphabet (Whitehurst & Lonigan, 1998). Together, these skills form the foundation for learning grapheme–phoneme correspondences.

Outside-in skills, on the other hand, encompass broader language abilities such as vocabulary, contextual understanding, and narrative skills. These abilities play a crucial role in the development of reading comprehension (Cabell et al., 2022; Gibson et al., 2021). A rich vocabulary, for example, enables children to grasp the meaning of spoken and written language across different contexts, thereby supporting overall language comprehension. Moreover, print concept knowledge is a foundational component of emergent literacy because it helps children understand how written language works before they begin formal reading instruction. It includes knowing that print carries meaning, recognizing the directionality of text (e.g., left to right, top to bottom), understanding the function of spaces and punctuation, and identifying basic features such as letters and words (Piasta et al., 2012).

Research shows that children with stronger print concept knowledge display faster growth in early reading skills, as these concepts allow them to orient themselves within text and more effectively link spoken and written language (Piasta et al., 2012). Acquiring print concepts in preschool thus provides a critical stepping stone toward successful literacy acquisition.

Early numeracy skills encompass the foundational mathematical competencies children acquire before the start of school, ranging from basic abilities, such as number symbol knowledge and simple counting, to more advanced skills including forward and backward counting from a specific number, number line estimation, and understanding of core mathematical principles like cardinality (Krajewski & Schneider, 2009; Nguyen et al., 2016). These skills form the basis for and act as strong predictors of mathematics achievement in primary school and beyond (Chang, 2023; Dierkx et al., 2025; Hornung et al., 2014; Jordan et al., 2007; Kleemans et al., 2012; Lehrl et al., 2020; Susperreguy et al., 2020).

Starting school with lower early literacy and numeracy skills puts children at a disadvantage that is unlikely to be fully compensated over time (Niklas, 2015). However, significant differences in literacy and numeracy skills among children can already be observed during preschool years due to specific characteristics (Niklas & Schneider, 2010). More advanced literacy skills tend to be found among girls, older children, those with higher intelligence, and children from families with higher SES and without a migration background (Mol et al., 2008; Peng et al., 2019). Similar patterns are observed for numeracy skills, which tend to be stronger among older children, those with higher intelligence, and children from families with higher SES and without a migration background (Anders et al., 2012; Hawes et al., 2019). Beyond family characteristics, such early differences can also be attributed to variations in children’s home learning environments (e.g., Daucourt et al., 2021; Niklas & Lehrl, 2025).

### 1.2. Home Literacy and Home Numeracy Environment

In his socioecological model, Bronfenbrenner (1979) ascribes an important role to a child’s closest social surroundings, most importantly their family. Through both proximal (e.g., parent–child interactions) and distal factors (e.g., SES), families, especially parents, can impact young children’s development. Role modelling and scaffolding support children in acquiring skills within a familiar framework (Burghardt et al., 2020). With the rising prominence of digital devices in families and young children’s lives, Johnson and Puplampu (2008) extended Bronfenbrenner’s model by a techno-subsystem nested within the microsystem, comprising children’s interactions with digital tools. This contemporary extension positions digital learning apps as proximal processes that run parallel and interact with parent–child interactions within the home, rather than as external add-ons to children’s everyday lives.

Within the broader family context, the home learning environment represents a key mechanism through which parental influences shape children’s developmental outcomes. Here, the home learning environment has been found to be particularly important in fostering and nurturing children’s abilities (e.g., Lehrl et al., 2020; Niklas & Schneider, 2017a; Susperreguy et al., 2020). It comprises resources and interactions that aim at supporting children’s learning and development. The richer the learning resources and the more active the parental engagement, the higher the quality of the home learning environment (Dearing et al., 2012). The general model of the home learning environment can be distinguished into different domains that target different abilities (Wirth et al., 2023). Great emphasis has been laid onto the HLE and the HNE that focus on parental affordances to support children’s emergent literacy and numeracy skills, respectively.

Both facets of the home learning environment include formal (e.g., teaching letters or numbers) and informal (e.g., shared reading, playing dice games) activities (Sénéchal & LeFevre, 2002; Susperreguy et al., 2020). For example, the HLE as a multifaceted construct also comprises two core dimensions: formal (code-related) and informal (meaning-related) activities. Formal activities involve explicit parental teaching, such as practicing letter recognition, phonological skills, name writing, and early word reading (Inoue et al., 2020; Krijnen et al., 2020; Torppa et al., 2022). In contrast, informal activities promote implicit literacy learning through everyday experiences and exposure to literacy-rich material. These informal experiences support the development of vocabulary, broader language skills, and later reading comprehension and fluency (e.g., Inoue et al., 2020; Lenhart et al., 2022; Torppa et al., 2022; see also a meta-analysis by Dowdall et al., 2020).

The HNE focuses on the frequency and quality of numeracy activities at home, parental support for children’s mathematical learning, as well as adequate materials available to the children (Mues et al., 2022; Niklas, 2015). The quality of the HNE is a significant predictor of children’s numeracy skills both in preschool and in primary school (Daucourt et al., 2021; Dearing et al., 2012; Mutaf-Yıldız et al., 2020; Napoli & Purpura, 2018; Silinskas et al., 2020). Specifically, formal (e.g., teaching how to count) and informal (e.g., dice games) activities target and significantly impact different facets of children’s numeracy skills (Elliott et al., 2017; Leyva, 2019; Silinskas et al., 2020; Susperreguy & Davis-Kean, 2016). For example, formal HNE activities predict children’s number symbol knowledge, whereas informal activities are rather associated with non-symbolic arithmetic (Skwarchuk et al., 2014).

### 1.3. Associations Between Child and Family Characteristics with the Home Learning Environment and Literacy and Numeracy Skills

Given that a child’s microsystem, that is, their family, is not isolated from other systems within Bronfenbrenner’s (1979) ecological model, the home learning environment is affected by various distant factors, especially child and family characteristics. Child characteristics include children’s age, sex and intelligence.

For instance, home learning activities can vary by sex, with parents often creating higher-quality HLEs for daughters and higher-quality HNEs for sons (Gustafsson et al., 2011; Vasilyeva et al., 2025; but see Mejía-Rodríguez et al., 2021). Accordingly, some studies show positive correlations between sex and the formal HLE, with girls living in a higher-quality formal HLE, and thus receiving more formal teaching (Novita & Kluczniok, 2022; Valcárcel Jiménez et al., 2025). Similarly, Mues et al. (2022) found both mothers and fathers to regard girls as less competent in numeracy than boys, and boys tended to have higher-quality HNEs than girls (Varnell et al., 2025; Vasilyeva et al., 2025). A possible explanation for this might be associated with gender stereotypes and child interest. For example, as parents of preschool children tend to rate girls higher on literacy interest (e.g., Baroody & Diamond, 2013), they might adapt the HLE they provide to their children (e.g., Højen et al., 2022), increasing the frequency of formal literacy teaching practices for daughters. The same reasoning could be applied to the HNE (cf. Varnell et al., 2025). Accordingly, some studies find greater literacy skills in girls (Lange et al., 2016; Lewicki et al., 2018; Vasilyeva et al., 2025); however, sex differences in numeracy skills do not seem to appear before the onset of formal schooling (Bakker et al., 2019; Kersey et al., 2018; Martinot et al., 2025).

Another important child characteristic for the development of early literacy and numeracy skills is intelligence. Research suggests that intelligence can support the acquisition of early literacy skills (e.g., Peng et al., 2019) and significantly predicts early numeracy skills (Coolen et al., 2021; De Smedt, 2022). Overall, while intelligence is linked to both early literacy and numeracy skills, it seems to be of greater importance for the latter (Kapteijns et al., 2025; Peng et al., 2019).

Beyond its direct association with literacy and numeracy skills, intelligence can also be associated with how children engage with and benefit from their home learning environment. Children with higher intelligence may find it easier to interpret prompts, symbols, or instructions embedded in literacy and numeracy activities, thereby potentially gaining more from the learning opportunities provided at home (e.g., Hadd & Rodgers, 2017).

In addition to child characteristics, family characteristics such as SES and migration background are of substantial importance for early literacy and numeracy development. The SES reflects a person’s social background and is typically defined by parental educational attainment and financial resources (Baumert & Maaz, 2006). It is considered one of the strongest determinants of academic achievement (Lee et al., 2011; Sonnenschein & Sun, 2017) and influences the HLE and children’s literacy skills (Jiang et al., 2024) as well as the HNE and children’s numeracy skills (DeFlorio & Beliakoff, 2015; Thompson et al., 2017). Here, consistent findings of lower-quality HLEs and HNEs among lower-SES families may be due to these parents being met with more (financial, social, or time-dependent) hurdles in providing appropriate learning materials or stimulating activities (Dearing et al., 2012). Accordingly, higher parental education and income predict more frequent, higher-quality parental engagement in home learning activities (Elliott & Bachman, 2018; Leyva, 2019), and higher-SES parents are more likely to engage in more informal HLE (Valcárcel Jiménez et al., 2025) and HNE activities (Elliott & Bachman, 2018; Susperreguy et al., 2020).

Such SES differences are reflected in children’s varying literacy and numeracy skill levels, with significant SES-related gaps already apparent before the start of formal schooling and only widening over the course of the school years in favor of children from higher-SES households (Jordan et al., 2007). Consequently, children from lower-SES households are expected to experience lower-quality HLEs and HNEs, which may limit the realization of their full skill potential. They require therefore additional support to receive similar learning opportunities as their higher-SES peers.

Research on the formal and informal components of the HLE among preschool children with a migration background is still emerging. Due to its strong connection with families’ SES (Autor:innengruppe Bildungsberichterstattung, 2024), it is difficult to untangle one from the other. Existing multigroup analyses indicate that, in Germany, children with a migration background tend to experience fewer informal literacy activities but engage more frequently in formal, instructional activities within the HLE (Novita & Kluczniok, 2022; Valcárcel Jiménez et al., 2024, 2025).

Furthermore, in many families with a migration background, German (i.e., the language of instruction) is not the primary language spoken at home. Instead, parents often use their mother tongue as the main language of communication (Novita & Kluczniok, 2022). As a result, children with a migration background may have limited exposure to German before entering preschool (Dubowy et al., 2008). This discrepancy between home and school languages adds complexity to understanding how the HLE supports literacy development in multilingual contexts. Parental engagement in home literacy activities may be shaped by the extent to which caregivers are familiar with the educational system their child is navigating, particularly when their own schooling took place in a different cultural context (Goodrich et al., 2025). Additionally, when the language of instruction differs from the language spoken at home, parents may face barriers to direct involvement in their children’s literacy development and broader educational experiences (Goodrich et al., 2025).

The literature review shows that children from different families receive learning opportunities in different frequencies, and that children from families who face structural disadvantages may particularly benefit from additional resources that strengthen their HLEs/HNEs to foster their literacy and numeracy skill development. The widespread availability of digital tools offers a promising means to address this need.

### 1.4. The Potential of Learning Apps Within the Home Learning Environment

With the increasing digitalization of everyday life, digital devices are also becoming part of young children’s home learning environments. Almost every family has access to the internet and smartphones, and three out of five children in Germany grow up in households that own at least one tablet (Medienpädagogischer Forschungsverbund Südwest (mpfs), 2024). According to the techno-subsystem extension of Bronfenbrenner’s socio-ecological model (Johnson & Puplampu, 2008), these digital devices lie not outside the home learning environment but are also embedded in proximal processes (e.g., parent–child interactions with, through, and about digital devices; media-child interactions). In this context, the digital home learning environment can serve as a complementary addition to the analog one, for example, through the use of learning apps (Lehrl et al., 2021).

As cultural tools and facilitators of social interactions, digital devices such as tablets can support young children’s knowledge acquisition. According to Vygotsky’s sociocultural theory (Vygotsky, 1978), higher cognitive functions develop through the mediation of interactions with more knowledgeable others within a child’s zone of proximal development. While this role was traditionally occupied by parents, the digitalization of everyday life brings digital devices to the foreground, reflecting the importance of the techno-subsystem (Johnson & Puplampu, 2008). By providing structured tasks, feedback, and adjustable difficulty, learning apps can function as mediational cultural tools that support children’s learning within proximal processes (Callaghan & Reich, 2020). With the help of learning apps, for example, children’s skills can be shaped and organized by the interaction with the device, if needed also without parental scaffolding (Falikman, 2021). Such apps offer flexible and stimulating learning opportunities, thereby adding a valuable digital dimension to the home learning environment (Lehrl et al., 2021; Niklas et al., 2025). Although research has demonstrated that learning apps have the potential to reduce educational gaps (Kim et al., 2021; Neumann & Neumann, 2017) and that digital interventions for literacy and numeracy development are at least equally effective compared to analog approaches (e.g., Aunio & Mononen, 2018; Barros et al., 2020; Dore et al., 2019; Dubé et al., 2019; Herodotou, 2018; Papadakis et al., 2018; Rachels & Rockinson-Szapkiw, 2017; Rogowsky et al., 2017; Vanbecelaere et al., 2020), little research has examined how learning apps may support children while considering the quality of the home learning environment they experience. Yet, such an investigation is particularly relevant, as it could inform approaches that accommodate parental constraints on time and financial resources while ensuring that children receive adequate developmental support. When the quality of the home learning environment is comparably low, the potential of digital devices should be particularly pronounced: Digital devices function as additional proximal processes within young children’s microsystems (Bronfenbrenner, 1979; Johnson & Puplampu, 2008) and can, to some extent, assume the role of more knowledgeable others in supporting children’s learning processes (Falikman, 2021; Vygotsky, 1978). Accordingly, well-designed learning apps may support children’s development, but their effectiveness likely depends on the quality of the home learning environment, such that they may be most beneficial when embedded in environments that lack frequent learning opportunities.

We refer to the term digital compensation as the capacity of high-quality digital learning experiences to compensate for limited or absent analog learning opportunities (Arnold et al., 2021). Under this framework, the use of digital learning apps has the potential to reduce, rather than reinforce, inequalities in early literacy and numeracy outcomes that arise from differences in the quality of the HLE and HNE.

### 1.5. The Current Study

The home learning environment plays a crucial role in young children’s cognitive development by providing learning resources, parental support, and cognitively stimulating activities that foster early literacy and numeracy skills (Bradley & Corwyn, 2002; Bronfenbrenner, 1979; Melhuish et al., 2008). However, the quality of both the HLE and HNE varies depending on child (e.g., sex, age, intelligence) and family (e.g., SES, migration background) characteristics (Mues et al., 2024; Valcárcel Jiménez et al., 2025). Digital tools such as learning apps may help reduce educational disparities by providing an affordable means of access to educational opportunities (Kim et al., 2021; Neumann & Neumann, 2017; Niklas et al., 2025; Schiele et al., 2025). However, these effects may depend on the quality of the HLE and HNE, respectively (Lehrl et al., 2021).

The current study therefore examines whether the quality of the HLE and HNE moderates the effectiveness of learning apps on preschool children’s literacy and numeracy gains. First, we expect that children in the literacy app intervention group recorded greater literacy skill gains than children from the other groups (H1a) and that children in the numeracy app intervention group recorded greater numeracy gains than children from the other groups (H1b). In both conditions (literacy and numeracy app intervention), we expect the differences to be more pronounced in comparison to the business-as-usual control group than to the tablet-control group, while still expecting an advantage over the latter due to the apps’ domain-specific educational content. Second, we hypothesize that longer literacy app usage will positively predict literacy outcomes six months later (H2a) and longer numeracy app usage will positively predict numeracy outcomes six months later (H2b). Finally, we expect that children with lower-quality HLEs will benefit more from literacy app usage (H3a), and children with lower-quality HNEs will benefit more from numeracy app usage (H3b), all while accounting for child and family characteristics.

## 2. Materials and Methods

Data were collected within the Learning4Kids study (Niklas et al., 2020, 2022). Families with children in the preliminary year of preschool living in the wider Munich area were invited to participate using address records from the Munich district administration, via outreach visits to local preschools, and by support of a professional recruiting company. Parents gave written consent for their own and their children’s participation. Ethical approval was granted by the ethics committee of the Faculty of Psychology and Educational Sciences at the University of Munich and conducted in accordance with the Declaration of Helsinki.

Overall, a total of *N* = 500 families were recruited (*M*_age_T1_ = 60.69 months, *SD* = 4.61, 257 girls). For the current study, the data of the first two measurement points (T1 and T2, approx. six months apart) were analyzed. Trained research assistants conducted home visits to assess children’s literacy and numeracy skills, among other competencies not of relevance for the current study.

The participants were randomly assigned to one of four conditions: (1) a literacy group (*n* = 151), receiving a tablet with literacy apps; (2) a numeracy group (*n* = 151), receiving a tablet with numeracy apps; (3) a tablet control group (*n* = 98), receiving a tablet with apps unrelated to literacy or numeracy (e.g., shapes, colors); (4) a business-as-usual control group (*n* = 100), receiving no tablet or apps.

Following T1 assessments, families in the tablet groups received a device, and children were briefly introduced to several apps. Children initially had access to four apps; additional new apps were automatically downloaded every four to five weeks to maintain engagement and ensure intervention fidelity. By the end of the intervention, children in the literacy group had access to 12 literacy apps (as well as 23 e-books) and children in the numeracy group had access to 18 numeracy apps (as well as five e-books).

Please see Table 1 for all descriptive statistics and reliability measures of all variables as well as Tables S1 and S2 in the supplemental material for the descriptive statistics of the literacy/numeracy groups only.

### 2.1. Literacy and Numeracy Apps

All learning apps were designed for preschool children and used a direct instruction approach (cf. Kebritchi & Hirumi, 2008). Simple touch-based navigation (e.g., tapping, dragging, tracing) was combined with verbal instructions and difficulty increased progressively across levels. In line with the four pillars of learning identified by Hirsh-Pasek et al. (2015), the apps were designed to be active, engaging, meaningful, and socially interactive, with opportunities for collaboration and competition. Some of the apps were evaluated by experts and received high ratings on the pillars (Wirth et al., 2024). The design principles placed a strong emphasis on avoiding background music and using child-friendly but non-distracting visuals (e.g., no flashing, no pop-ups; Mayer, 2014). The learning elements of the apps focused strongly on feedback (Bai et al., 2020), which was provided verbally (i.e., positive feedback if the answer was correct, encouraging feedback to try again if the answer was incorrect), visually (e.g., stars, balloons for correct answers), auditorily (e.g., cheering, clapping), or through badges (e.g., number of stars achieved after completing a level depending on the number of errors made).

Eleven of the twelve literacy apps used were developed within the Learning4Kids study (Table S3), with the addition of one app freely available in the play stores in the final month. The apps trained early literacy skills including letter learning, phonological awareness, word and sentence comprehension, and letter tracing/sorting.

Seventeen of the 18 numeracy apps used were developed within the Learning4Kids study and one was freely available in the play stores (Table S4). They targeted early numeracy skills such as number recognition, sequencing, sorting, measurement, arithmetic, and time concepts.

Children’s engagement with the apps was tracked using mobile sensing technology (Birtwistle et al., 2022). The cumulative time spent across all literacy or numeracy apps was computed as “Literacy App Usage Time” (LAUT) and “Numeracy App Usage Time” (NAUT), respectively. Z-standardized scores of the two variables were used in the analyses to facilitate interpretation of models with interaction terms (Aiken & West, 1991).

### 2.2. Home Learning Environment Measurements

We assessed both aspects of the home learning environment via parental self-reports on paper-pencil surveys at T1 (see Supplementary S1 in the Supplementary Materials).

The HLE was measured using 24 items. Parents indicated how often they or their partner read to their child (4 = several times a day to 0 = less often/never), the amount of (children’s) books in their house (4 = more than 100 children’s books/200 books to 0 = none), and various formal (e.g., teaching the alphabet) and informal home literacy activities (e.g., shared reading) on 5-point Likert scales (4 = several times a day to 0 = less often/never; e.g., “How often do you sing with your child or recite rhymes?”). Composite scores of all items formed the variable “HLE” for the analyses.

The HNE comprised 12 items. Parents reported on the frequency of their formal (e.g., practicing numbers) and informal (e.g., playing dice games) home numeracy activities on 5-point Likert scales (4 = several times a day to 0 = less often/never; e.g., “How often do you play dice games with your child?”). Composite scores of all items formed the variable “HNE” for the analyses.

### 2.3. Child Measurements

Literacy skills were assessed at both T1 and T2 using several subtests from three standardized instruments. Early literacy knowledge was measured with a subtest of the Assessment of narrative and reading competencies of 4- to 5-year-old children (EuLe; Meindl & Jungmann, 2019). Children were presented with a picture book containing simple sentences and were asked to recognize features of literacy, such as capital letters or reading direction (correct = 1, incorrect = 0). For the analysis, sum scores were calculated.

Phonological awareness, plural formation, and active and passive letter knowledge were assessed using five subtests from the Würzburg preschool test (WVT; Endlich et al., 2017). Two tasks were used to measure phonological awareness. First, children were required to identify the one word that did not rhyme with the others among four words (e.g., “See”—“Tee”—“Tisch”—“Klee”). Second, children named the initial sound of depicted objects (e.g., /l/ for “Laster” [truck]). For the plural formation task, children were shown pictures of fictitious creatures with names resembling actual German words (e.g., “Naus”, resembling “Maus” [mouse]). The children were asked to produce the correct plural form (e.g., “Näuse”, resembling “Mäuse” [mice]). Passive and active letter knowledge was measured through letter recognition. Children identified specific letters among four options (e.g., “Please show me /r/.”). Additionally, children actively named a presented letter (e.g., “N”). For all subtests, correct responses were coded as “1”, incorrect responses as “0”. Sum scores were computed for the analyses.

Receptive vocabulary was assessed with nine sets of the German version of the Peabody Picture Vocabulary Test (PPVT; Lenhard et al., 2015). There were 12 items in each set, each with four pictures. Children pointed to the corresponding image after hearing a target word (e.g., “hedge”) (correct = 1, incorrect = 0). The final sum score was calculated by subtracting incorrect responses from the total number of items. All sum scores were z-standardized, as maximum scores differed between the literacy measures.

Numeracy skills were assessed at T1 and T2 using adjustments of standardized tests. Knowledge on cardinality, number division and relations, multiplication, addition, and subtraction were assessed with subtests of the Mathematics and Calculation Concepts in Preschool Age Screening (MARKO-S; Ehlert et al., 2020) on 21 items. Knowledge on numbers, number sequences forward and backward, and number predecessors and successors were assessed with four subtests of the WVT (Endlich et al., 2017) on six to 10 items per subtest. Arithmetic skills were assessed with an adapted version of the Test of Mathematical Basic Competencies in Kindergarten Age (MBK-0; Krajewski, 2018) on eight items. All correct responses were coded with “1”, all incorrect responses with “0”. Sum scores of each subtest were calculated and z-standardized.

In addition to literacy and numeracy skills, we controlled for children’s passive screen time, reported by their parents at T1. On a 5-point Likert scale, parents indicated how much time their children spent with digital media on average per week: more than 25 h per week (4), 10–25 h per week (3), 5–10 h per week (2), 1–5 h per week (1), or less than 1 h per week (0).

### 2.4. Child and Family Characteristics

Non-verbal intelligence was measured at T2 via the Columbia Mental Maturity Scale (CMMS; Burgemeister et al., 1972) across 57 items. Children identified the odd object out of five (e.g., four forks, one spoon; correct = 1, incorrect = 0).

Children’s age was controlled for the analyses. Socioeconomic status was assessed via family income, parents’ highest educational attainment, and the highest prestige value of parental occupation (Wegener, 1988), as reported by parents at T1. The prestige scale ranks 283 categories of the International Standard Classification of Occupations (ISCO) based on their socially ascribed prestige. The scale ranges from 20 (unemployed) as the lowest value to 186.8 (physician) as the highest value, and our sample encompassed the entire range. All indicators of SES were z-standardized.

Children who spoke primarily a different language than the instruction language (i.e., German) at home were coded as having a migration background (38% in our sample). This proportion of children with migration background is similar to the proportion of approximately 43% for children between five and ten years old in Germany (as of 2025; Federal Statistical Office, 2025).

### 2.5. Analytical Approach

Analyses were conducted using R 4.3.1 (R Core Team, 2025). We decided to replace observations with extremely high average app usage times with missing values, based on a statistical criterion of values exceeding +5 SD (LAUT: *n* = 1; NAUT: *n* = 3). Such values were considered implausible indicators of active app engagement and more likely reflected recording artifacts (e.g., passive screen time) than actual usage. Two outliers with a score lower than 10 out of 57 on the non-verbal intelligence test were excluded from the analyses. Complete cases analysis would have consisted of *n* = 358 children, resulting in a loss of roughly 28% of the data. Therefore, missing values were imputed using multiple imputation by chained equation (mice package, Van Buuren & Groothuis-Oudshoorn, 2011). All variables were z-standardized for multiple imputation.

Both descriptively (see Figure S1) and inferentially, quadratic effects emerged for the relation between literacy skills at T2 and LAUT (*p* < 0.001, adj. R^2^_linear_ = 0.01 vs. adj. R^2^_quadratic_ = 0.04). For the relation between numeracy skills at T2 and NAUT, no significant quadratic effects were detected (see Figure S1; *p* = 0.104, adj. R^2^_linear_ = 0.001, adj. R^2^_quadratic_ = 0.003). However, to maintain parallel analytic procedures across domains, quadratic terms were computed for both LAUT and NAUT. This resulted in the variables LAUT_sq and NAUT_sq. To avoid underestimating potential quadratic effects and interaction terms in the imputed datasets, interaction terms were created prior to imputation separately for the literacy and numeracy groups: For the literacy model, interactions between HLE and both LAUT and LAUT_sq were specified, and for the numeracy model, interactions between HNE and both NAUT and NAUT_sq were specified. Literacy skills at T2 were reported as quadratic outcome of LAUT and numeracy skills at T2 were reported as quadratic outcome of NAUT (Vink & van Buuren, 2013). Through this approach, we generated 100 imputed datasets (e.g., Graham et al., 2007) using all key variables to investigate the hypotheses. The datasets were generated using predictive mean matching for continuous variables, logistic regression for dichotomous variables, and the quadratic effects were handled following the approach proposed by Vink and van Buuren (2013). For each hypothesis, regression analyses were conducted across all 100 imputed datasets, and the estimates were pooled based on Rubin’s rules (Schafer & Olsen, 1998).

In a first confirmatory analysis, we examined whether children differed in their baseline literacy/numeracy skills based on their HLE/HNE at T1. For this, literacy/numeracy skills at T1 were regressed on HLE/HNE at T1.

For H1a, we set up two linear regression models. The first model regressed literacy skills at T2 on prior knowledge and the dummy-coded group variable (literacy/numeracy/tablet-control/control). The second model added the control variables sex, age, intelligence, SES, migration background, and passive screen time. Estimated marginal means and planned pairwise contrasts were derived for each imputed data set from the regression models using Rubin’s rules to test whether the literacy group outperformed the comparison groups. Additional contrast tests examined whether the potential literacy advantage was significantly larger relative to the business-as-usual control group relative to the numeracy or tablet-control groups.

For H1b, the procedure was the same, with the first model regressing numeracy skills at T2 on prior knowledge and group and the second model adding all control variables. Additional contrast tests examined whether the potential numeracy advantage was significantly larger relative to the business-as-usual control group relative to the literacy or tablet-control groups. Power sensitivity analyses indicated that effects of at least f^2^ = 0.03 (small effect) should be detectable (α = 0.05, power = 0.90, *n* = 500).

For H2a, two linear regression models were set up. Within a subset of children who belonged to the literacy app intervention group (*n* = 151), the first model regressed literacy skills at T2 on prior knowledge (i.e., literacy skills at T1), LAUT, and LAUT_sq. The second model added the control variables. For H2b, another two regression models were conducted. Within a subset of children who belonged to the numeracy app intervention group (*n* = 151), the first model regressed numeracy skills at T2 on prior knowledge (i.e., numeracy skills at T1), NAUT, and NAUT_sq, and the second model added the control variables. Power sensitivity analyses indicated that effects of at least f^2^ = 0.09 (small to medium effect) should be detectable (α = 0.05, power = 0.90, *n* = 151).

For H3a, four regression models were set up, using a subset of children who belonged to the literacy app intervention group (*n* = 151). First, baseline-corrected literacy gains were modeled controlling for literacy skills at T1. Second, LAUT, LAUT_sq, HLE, and the control variable passive screen time were added. Third, interaction terms between HLE and LAUT as well as HLE and LAUT_sq were added. Finally, this model was extended by all control variables.

For H3b, the same procedure was used, with 100 imputed datasets investigating a subset of children who belonged to the numeracy app intervention group (*n* = 151). Four regression models were set up. First, baseline-corrected numeracy gains were modeled controlling for numeracy skills at T1. Second, NAUT, NAUT_sq, HNE, and the control variable passive screen time were added. Third, interaction terms between HNE and NAUT as well as HNE and NAUT_sq were added. Finally, this model was extended by all control variables.

Power sensitivity analyses indicated that effects of at least f^2^ = 0.10 (small to medium effect) should be detectable (α = 0.05, power = 0.90, *n* = 151). Models analyzed for H3a and H3b were compared based on the Akaike Information Criterion (AIC; lower values indicate greater predictive value), Bayesian Information Criterion (BIC; lower values favor more parsimonious models), and sample-size-adjusted BIC (aBIC; indicating intermediate penalty between AIC and BIC; lower values preferred).

## 3. Results

### 3.1. Descriptive Analyses

Table 2 depicts the correlation analysis between all study variables. Literacy and numeracy skills were strongly correlated at both T1 and T2, both within and between constructs. Both skills were also significantly associated with both HLE and HNE. Children with better literacy and numeracy skills at T1 thus showed greater literacy and numeracy skills at both T1 and T2 and lived in higher-quality HLEs and HNEs. Older children, children with greater intelligence, from higher-SES backgrounds, and without a migration background showed greater literacy and numeracy skills at T1 and T2 than their peers. While no significant sex differences were found in literacy skills, boys recorded greater numeracy skills at T1 and T2. Literacy skills at T1 and HLE were correlated with children’s passive screen time, indicating that greater skills and a higher-quality HLE were associated with longer weekly media usage. Literacy skills at T2 were significantly correlated with LAUT, indicating that greater skills were associated with longer usage time of the literacy apps. Numeracy skills and NAUT, however, were not significantly correlated.

A higher-quality HLE was significantly correlated with a higher-quality HNE. Younger children, those with greater intelligence, from higher-SES families, and without a migration background lived in a higher-quality HLE, whereas only intelligence and SES were significantly and positively correlated with a higher-quality HNE. Neither HLE nor HNE were associated significantly with literacy/numeracy app usage times.

Finally, children with higher intelligence scores were more likely to be girls, from higher-SES households, and without a migration background. Children from higher-SES households were younger, rather without a migration background, and had more frequent weekly media usage.

Overall, the correlations showed a strong interconnection between literacy and numeracy skills as well as between HLE and HNE, and the relevance of child and family characteristics for both skills and home learning environments. The app usage times seemed unrelated to most of the study variables.

### 3.2. Pre-Analysis: Differences in Baseline Literacy/Numeracy Skills Based on Home Literacy/Numeracy Environment

Linear regression models revealed HLE as significant predictor of baseline literacy skills, with increasing HLE levels predicting greater literacy skills (β = 0.27, *SE* = 0.04, *F*(421.80) = 6.29, *p* < 0.001). Similarly, children with higher HNE levels showed greater numeracy skills at T1 than their peers (β = 0.20, *SE* = 0.04, *F*(484.66) = 4.59, *p* < 0.001).

### 3.3. Differences in Literacy/Numeracy Skill Gains Depending on Intervention Group Membership

**Figure 1:**
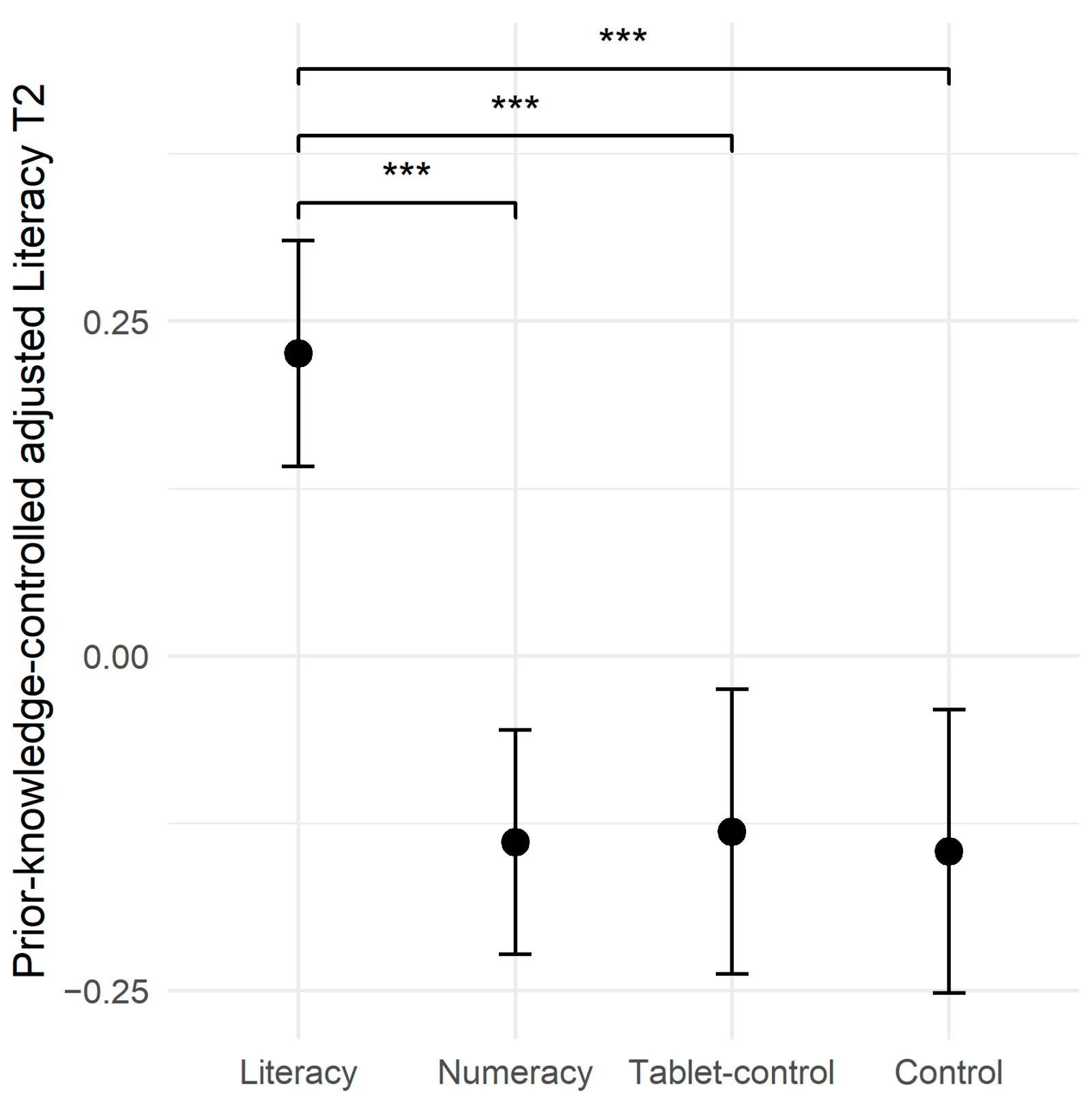
Literacy skill means at T2 by intervention group, controlled for prior knowledge and child sex, age, intelligence, passive screen time, and family socioeconomic status and migration background. *** *p* < 0.001.

Considering intervention group membership in predicting literacy skill gains (H1a) showed that children who did not receive the literacy app intervention recorded significantly lower literacy skills at T2 compared to the literacy app intervention group, after controlling for prior knowledge (Table S5, Model A1). This was true even after controlling for sex, age, intelligence, SES, migration background, and passive screen time (Model A2). Planned comparisons based on estimated marginal means (see Figure 1) showed that children from the literacy group showed greater literacy skills at T2 than children in the numeracy group (Δ_Num-Lit_ = −0.36, *SE* = 0.06, *t*(99) = −6.01, *p* < 0.001), tablet-control group (Δ_T-C-Lit_ = −0.36, *SE* = 0.07, *t*(99) = −5.22, *p* < 0.001), and control group (Δ_Con-Lit_ = −0.38, *SE* = 0.07, *t*(99) = −5.40, *p* < 0.001). The literacy advantage relative to the control group compared to the advantage relative to the numeracy group was not significant (Δ_Lit_vs_Con-Lit_vs_Num_ = −0.01, *SE* = 0.09, *t*(99) = −0.08, *p* = 0.938). Similarly, the literacy advantage relative to the control group compared to the advantage relative to the tablet-control group was not significant (Δ_Lit_vs_Con-Lit_vs_T-C_ = −0.01, *SE* = 0.10, *t*(99) = −0.13, *p* = 0.899). This means that the literacy intervention did not show a greater benefit compared to the control group than it did compared to the other two comparison groups (numeracy and tablet-control).

**Figure 2:**
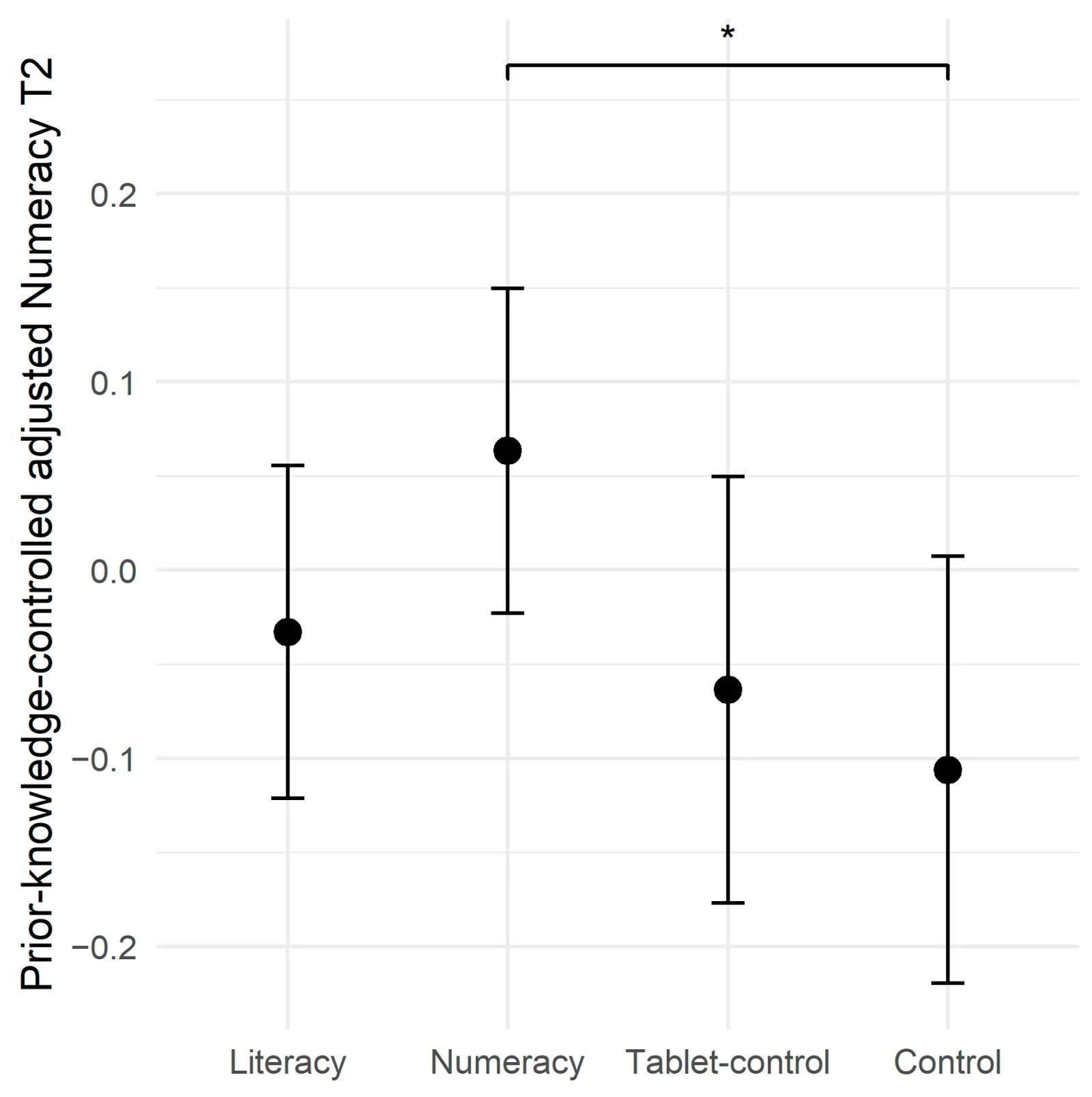
Numeracy skill means at T2 by intervention group, controlled for prior knowledge and child sex, age, intelligence, passive screen time, and family socioeconomic status and migration background. * *p* < 0.05.

In contrast, children who received the numeracy app intervention scored higher on numeracy skill outcomes at T2 than the other groups, after controlling for prior knowledge, but this finding reached only significance for the comparison with the business-as-usual control group (*p* = 0.008; for literacy group *p* = 0.051; for tablet-control group *p* = 0.060; see Model B1). The same was true after controlling for child and family characteristics (Model B2). Planned comparisons based on estimated marginal means (see Figure 2) showed that children from the numeracy group scored descriptively but not significantly greater numeracy scores at T2 than children in the literacy group (Δ_Lit-Num_ = −0.10, *SE* = 0.06, *t*(99) = −1.57, *p* = 0.119) and tablet-control group (Δ_T-C-Num_ = −0.13 *SE* = 0.07, *t*(99) = −1.79, *p* = 0.077). Significant differences were found in comparison to the control group (Δ_Con-Num_ = −0.17, *SE* = 0.07, *t*(99) = −2.36, *p* = 0.020). The numeracy advantage relative to the control group compared to the advantage relative to the literacy group was not significant (Δ_Num_vs_Con-Num_vs_Lit_ = −0.07, *SE* = 0.09, *t*(99) = −0.08, *p* = 0.936). Similarly, the numeracy advantage relative to the control group compared to the advantage relative to the tablet-control group was not significant (Δ_Num_vs_Con-Num_vs_T-C_ = −0.04, *SE* = 0.10, *t*(99) = −0.13, *p* = 0.897). This means that the numeracy intervention did not show a significantly greater benefit compared to the control group than it did compared to the other two comparison groups (literacy and tablet-control).

Taken together, we found that children in the literacy app intervention group recorded significantly greater literacy skill gains than those from all other groups (H1a). However, gains were not more pronounced in comparison to the business-as-usual control group than to the tablet-control group. For children in the numeracy app intervention group, significantly greater numeracy skill gains were found compared to the no-tablet control group; however, no significant differences were observed compared to the literacy or tablet-control group (H1b, but *p* < 0.10). Again, the differences of the three comparisons were not more pronounced in any comparison.

### 3.4. Predictiveness of Longer App Usage for Literacy/Numeracy Skill Gains

**Figure 3:**
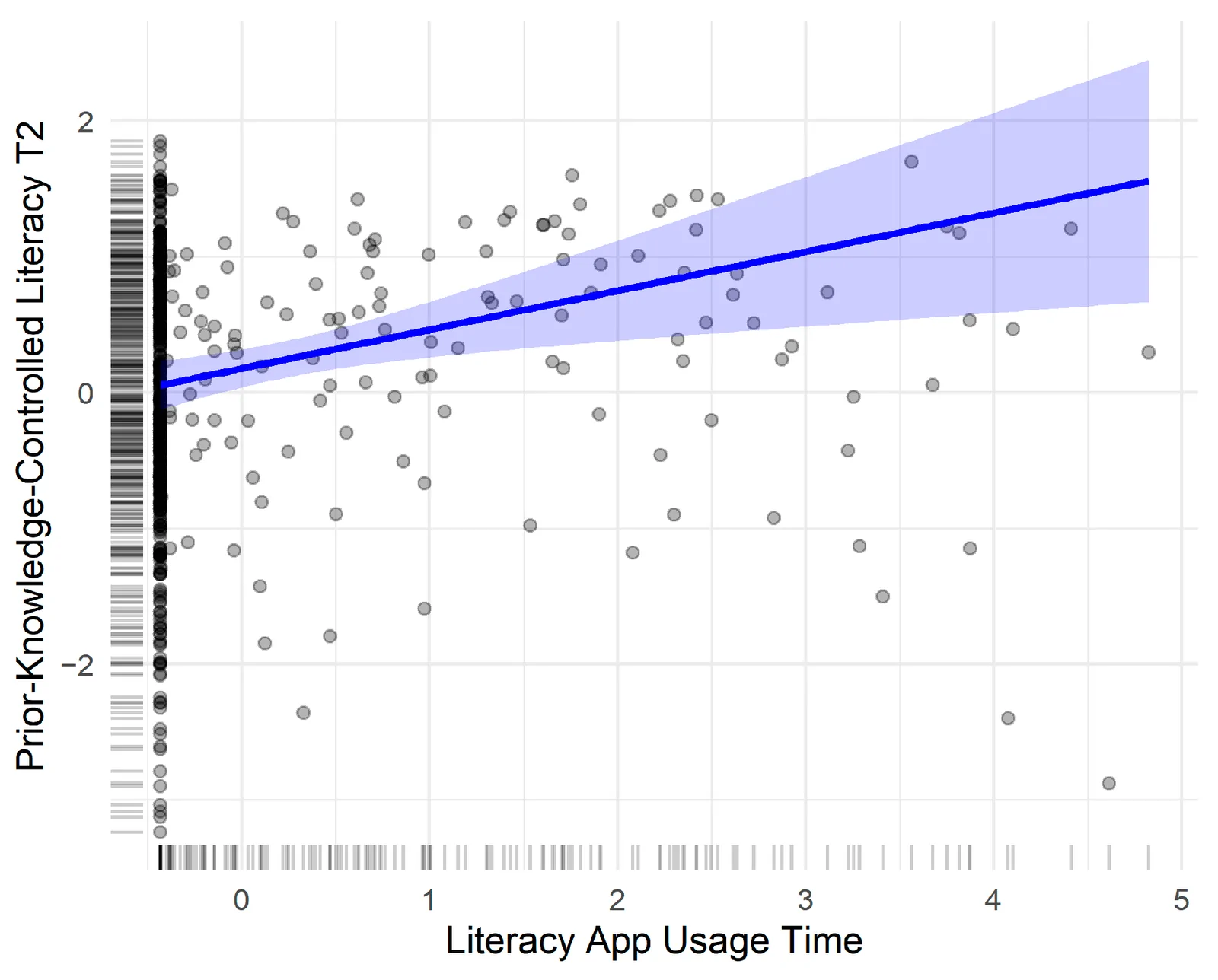
Regression of literacy app usage time on literacy gains, controlling for child sex, age, intelligence, passive screen time, family SES, and migration background.

Regression models with a subset of the literacy app intervention group (H2a; see Table S6, Model A1) showed significant predictive paths from LAUT to literacy skill outcomes at T2, indicating that children who played longer with the literacy apps recorded greater literacy gains (β = 0.34, *p* < 0.001). This effect did not diminish when controlling for prior knowledge and child and family characteristics (see Figure 3, β = 0.29, *p* = 0.003). Additionally, next to prior knowledge (β = 0.62, *p* < 0.001), LAUT_sq (β = 0.00, *p* = 0.016), and SES (β = 0.18, *p* = 0.002) were significant predictors for literacy skills at T2 (Model A2).

**Figure 4:**
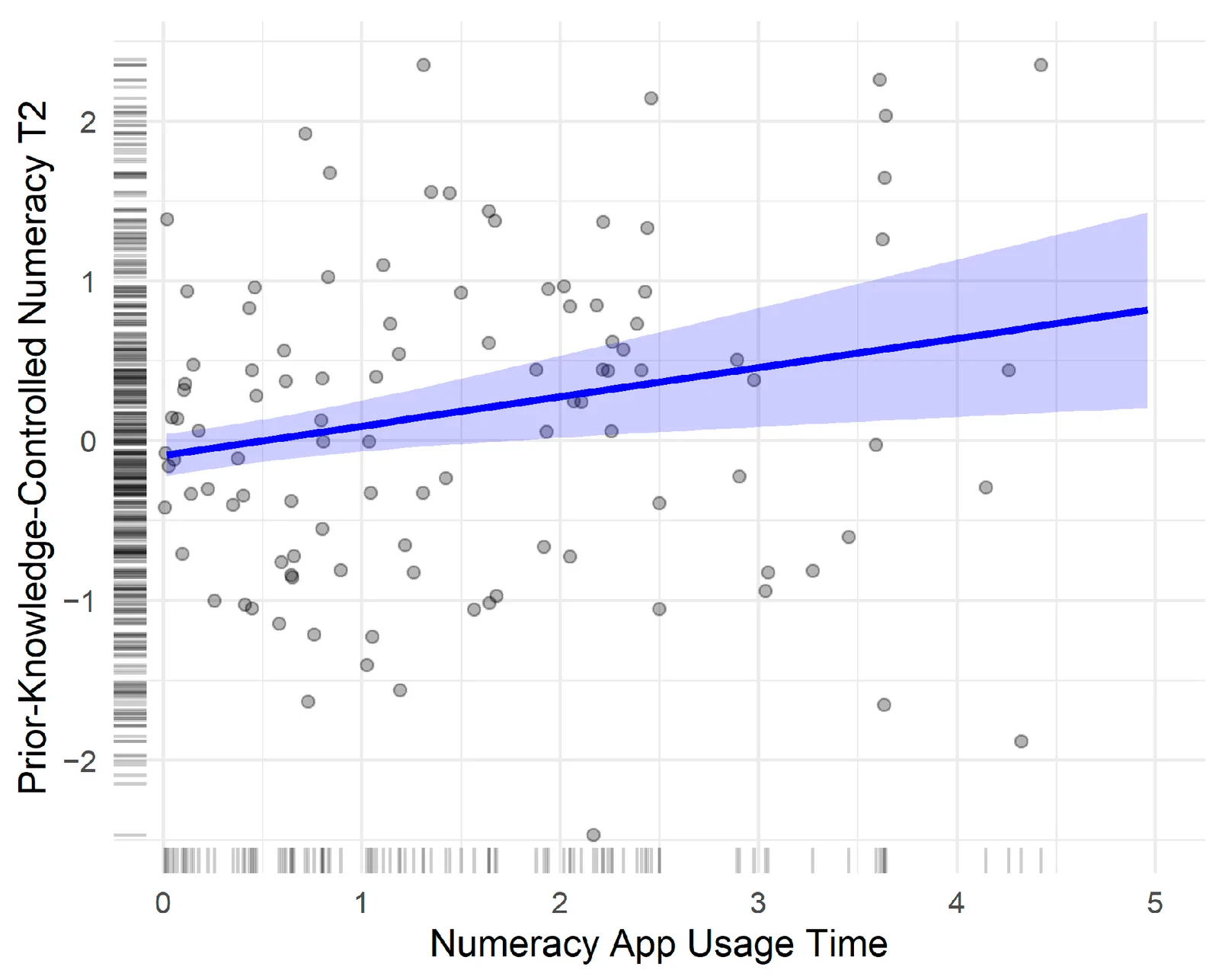
Regression of numeracy app usage time on numeracy gains, controlling for child sex, age, intelligence, passive screen time, family SES, and migration background.

Regression models within the numeracy app sample (H2b; see Table S6, Model B1) revealed NAUT as a significant predictor of numeracy skill outcomes at T2, meaning that children who played longer with the numeracy apps recorded greater numeracy gains than their peers who played less with the apps (β = 0.21, *p* = 0.003). This effect did not diminish when controlling for prior knowledge and child and family characteristics (see Figure 4, β = 0.18, *p* = 0.005). Additionally, next to prior knowledge (β = 0.67, *p* < 0.001), NAUT_sq (β = 0.00, *p* = 0.044), intelligence (β = 0.20, *p* = 0.001), and SES (β = 0.11, *p* = 0.033) were significant predictors of numeracy skills at T2 (Model B2).

Taken together, we found that longer literacy app usage positively predicted literacy outcomes six months later (H2a) and that longer numeracy app usage positively predicted numeracy outcomes six months later (H2b).

### 3.5. Interaction Terms Between HLE/HNE and App Usage to Predict Literacy/Numeracy Gains

To investigate whether children with lower HLE levels would benefit more from literacy app usage (H3a), we set up multiple linear regression models within a subset of children who received the literacy app intervention (*n* = 151). The results of all four models can be seen in Table S7. Model-comparisons statistics for all four models can be seen in Table S8 and Figure S2. Based on its predictive value and theoretical relevance, the results of the full model, including all control variables (Model A4), will be reported in the following. No significant interaction effects between HLE and LAUT (β = -.05, *p* = 0.670) or HLE and LAUT_sq (β = 0.00, *p* = 0.940) were found. On their own, LAUT (β = 0.30, *p* = 0.003) and LAUT_sq (β = 0.00, *p* = 0.017) were significant predictors of literacy gains. Although a tendency was found, HLE did not emerge as a significant predictor of literacy skill gains between T1 and T2 (β = 0.12, *p* = 0.051). Additionally, prior knowledge (β = 0.60, *p* < 0.001) and SES (β = 0.14, *p* = 0.021) were significant predictors of literacy outcomes at T2.

Another set of multiple regression models was set up to investigate whether children with lower HNE levels would benefit more from numeracy app usage (H3b). The results of all four models can be seen in Table S9. Model-comparison statistics for all four models can be seen in Table S8 and Figure S2. Based on model comparison and its explained variance and theoretical relevance, the results of the full model, including all control variables (Model B4), will be reported in the following. There were no significant interaction effects between either HNE and NAUT (β = 0.04, *p* = 0.455) or HNE and NAUT_sq (β = 0.00, *p* = 0.450). Whereas NAUT (β = 0.18, *p* = 0.005) and NAUT_sq (β = 0.00, *p* = 0.045) were significant predictors for numeracy skill gains, HNE was not (β = 0.00, *p* = 0.988). Prior knowledge (β = 0.67, *p* < 0.001), intelligence (β = 0.22, *p* = 0.001), and SES (β = 0.11, *p* = 0.029) were significant predictors of numeracy outcomes at T2.

Taken together, we found that children with less stimulating HLEs did not benefit more from literacy app usage (H3a), and children with less stimulating HNEs did not benefit more from numeracy app usage (H3b).

## 4. Discussion

This study investigated whether educational learning apps can support children’s literacy/numeracy skill gains compared to children who did not receive literacy/numeracy apps. Additionally, it was investigated whether learning apps can compensate for lower-quality HLEs/HNEs in preschool children’s literacy/numeracy gains. Although the use of learning apps developed within this study was a significant predictor of the development of children’s literacy/numeracy skills, it showed no significant interaction with families’ HLE/HNE, thereby putting into question to what extent learning apps can compensate for lower-quality learning environment.

### 4.1. Children in the Intervention Groups Show Significantly Greater Literacy/Numeracy Gains than Children in the Control Groups

As expected, children who received the literacy app intervention recorded significantly greater literacy gains than those in the other groups; similarly, children who received the numeracy app intervention recorded greater numeracy gains than others, but this effect reached significance only when compared to the business-as-usual control group. The findings extend the current body of research on the effectiveness of digital learning interventions for young children (Kim et al., 2021; Neumann & Neumann, 2017). However, the gains were not significantly greater in the intervention groups compared to the business-as-usual control group relative to the other comparison groups, neither in the literacy nor the numeracy group. In other words, children who did not receive any learning apps did not show significantly lower skill gains compared to the groups of children who received learning apps covering other domains. This suggests that it seems less important whether there is any interaction with a digital device at all, and that rather the content is key (see also Clemente-Suárez et al., 2024).

Interestingly, stronger effects emerged for the literacy group than for the numeracy group, even though children on average spent less time playing the literacy apps compared to the numeracy apps. This finding reflects previous findings indicating that the effectiveness of literacy apps is much better established than that of numeracy apps (Wirth et al., 2024). Notably, quadratic effects appeared for the relations between skill gains and app usage time: Beyond a certain average playtime, the beneficial effect of the apps seemed to reverse, and children who played more than 48 min per week (seven minutes per day) showed, at least descriptively, smaller literacy gains (see Figure S1). Prior research has documented the negative effects of excessive media usage on children’s development (Dore et al., 2019), but the app usage time in the current study can hardly be compared to the excessive media usage time of more than four hours per day reported by Dore et al. In contrast, far less is known about the other end of the spectrum of media usage—that is, how little media usage is actually needed before any effect can be detected at all. Within our sample, already five to seven minutes per day seemed beneficial in supporting literacy and numeracy skill gains (see also Niklas et al., 2025; Schiele et al., 2025). At the same time, the findings also indicate that, among the children who played with our apps, more was not necessarily better: Past a certain threshold, additional app usage may have no longer translated into additional skill gains and could even have had counterproductive effects for some children. However, as app usage duration was not randomly assigned, our dose–response finding should not be interpreted causally.

While children in the literacy intervention group showed significantly greater skill gains than children in the comparison groups, this was only true for children in the numeracy intervention group relative to the business-as-usual control group—that is, compared only to children who received no apps at all. It is possible that the provided learning apps fostered general cognitive abilities (i.e., across all tablet groups) that are also relevant for numeracy skills such that the focus on literacy or on shapes and colors similarly supported some of the cognitive abilities underlying numeracy skills (e.g., Nur et al., 2022).

Overall, the findings indicate that high-quality learning apps are beneficial (Wirth et al., 2024) and can be meaningfully used to support children’s learning. The exact mechanisms spurring this as well as the question which app and which design works best for whom still need to be worked out.

### 4.2. Longer App Usage Time Predicts Literacy and Numeracy Skill Gains

In line with our expectations, we found that educational learning apps developed to support children’s literacy and numeracy learning significantly predicted literacy and numeracy gains. Children who played longer with the learning apps recorded greater literacy/numeracy gains on average than those who played less with the apps. This finding extends literature on the beneficial impact of learning apps on children’s academic skill development (e.g., Bai et al., 2020; Berkowitz et al., 2015; Kim et al., 2021; Neumann & Neumann, 2017; Papadakis et al., 2018; Xie et al., 2018) and provides further evidence that apps can be supportive of children’s development if they are conceptualized according to pedagogical standards (Hirsh-Pasek et al., 2015). Such apps can thus be an additional opportunity to support young children’s skill development, in addition to support from their analog learning environments, independent of child and family characteristics (see also Niklas et al., 2025; Schiele et al., 2025). As such, learning apps may serve as a valuable resource in contexts where time or material resources are limited, for example in preschool or at home.

However, longer app usage time may also be confounded by other variables such as interest or parental involvement. Children who engaged more with the apps and thus recorded greater skill gains on average might have done so because they were interested in literacy/numeracy activities anyway, and similar findings could have occurred with analog interventions as well. App usage for these children might have also been encouraged and accompanied by parents, which could have created a synthesis of digital and parental mediation (cf. Falikman, 2021). As only quantitative data were collected, no conclusions can be drawn about the children’s interest in or the nature of their engagement with the apps. Nevertheless, learning apps can also help foster existing interests or—thanks to their motivational design—spark new ones in the first place (Amaefule et al., 2023). They can also provide new impetus for parent–child interactions and, for example, help parents effectively scaffold their children’s learning processes (e.g., Rowe et al., 2021).

### 4.3. Absence of Moderation Effects Indicate Limited Digital Compensation for Lower-Quality Home Learning Environments

Against expectations, we did not find a significant interaction between learning apps and the home learning environment on children’s literacy/numeracy skill gains. However, this null finding should be interpreted with caution, as the observed effects were substantially smaller than those detected within a sensitivity analysis (see Section 2.5). With this caveat in mind, our learning apps do not appear to unfold compensating effects for lower-quality home learning environments. In fact, when considering the interaction between app usage times and home learning environment, only LAUT and NAUT as well as their squared terms but not HLE/HNE significantly predicted literacy/numeracy skill gains anymore.

One possible explanation for this finding could be that the variance in skill gains attributable to the HLE/HNE overlaps with the variance captured by children’s engagement with the learning apps, meaning that the HLE/HNE on their own contributed little once app usage times were considered. Since the content of the learning apps and assessment instruments was largely aligned, it is also possible that longer app usage time better prepared for the assessments than the relatively more coarse-grained HLE/HNE. Additionally, an even greater overlap in the variance explained by the HLE occurred probably with the variances captured by intelligence and SES, considering the moderate correlations between HLE and these variables.

While the HNE appeared to have virtually no predictive effect (β = 0.00, *p* = 0.988), marginal effects were found for the HLE (β = 0.12, *p* = 0.051). The HLE may therefore retain greater relative importance for children’s literacy competencies development than the HNE for children’s numeracy competencies development, even when children additionally use learning apps. This may be because the HLE is more deeply embedded in social interactions and routines, such as shared reading, conversations, or exposure to print. These activities are interactive and adaptive, characterized by contingent feedback, elaboration, and language adapted to children’s needs (e.g., Georgiou et al., 2021; Silinskas et al., 2020). In contrast, the learning apps used in the current study represent a more structured format that is limited in its ability to adapt to children’s individual levels and spontaneous needs (e.g., questions). Consequently, it is possible that the HLE provides qualitatively richer learning experiences, which could explain its (albeit marginal) predictive value for literacy outcomes. In fact, sensitivity analyses including extreme values in app usage time yielded a significant association between HLE and literacy skill gains (β = 0.16, *p* = 0.006), while LAUT and LAUT_sq were no longer significant predictors. Although this finding should be interpreted cautiously, given the exploratory nature of the analysis and differences in the treatment of extreme values, the findings provide additional support for the relevance of HLE for literacy development, even when considering educational learning apps.

On the other hand, even though parents engage frequently in home numeracy activities, as our descriptive data suggests, they may scaffold children’s numeracy learning differently to their literacy learning. Research suggests that parents often feel more confident engaging in literacy activities than in numerical ones (Bosire et al., 2025; Cannon & Ginsburg, 2008; Uscianowski et al., 2020). Therefore, parents may provide less elaborated explanations, fewer prompts, or less adaptive support when engaging in numeracy-related interactions. Consequently, even when numeracy activities occur at home, they may not consistently translate into measurable gains in children’s numeracy skills. However, it is important to consider the asymmetrical measurement of HLE versus HNE when interpreting the findings. The HNE was assessed with half as many items as the HLE (12 vs. 24), meaning that the HNE inventory sampled a narrower range of home numeracy activities. This may attenuate the scale’s association with numeracy skill gains. It is therefore unclear to what extent the differential findings for HLE versus HNE observed here reflect a genuine difference in how parents scaffold literacy versus numeracy learning, as opposed to a difference in how comprehensively the two constructs were measured.

Across both literacy and numeracy domains, the interaction between app usage times and HLE/HNE did not significantly predict literacy/numeracy outcomes. These results indicate no evidence that apps compensate for qualitatively weaker home learning environments or add to the benefit of already strong ones. Regarding the results of the effectiveness of our apps compared to the business-as-usual control group, the findings point to supplementing rather than compensating features of learning apps.

Furthermore, it may be that the current findings are due to the intervention’s focus on children without explicitly involving the parents as well. Specifically in lower-quality home learning environments, parents can benefit from trainings on how to support their children, including how to use learning apps adequately and as a means to foster their children’s skill development (e.g., Niklas & Schneider, 2017b). Without engaging parents, children may learn from the apps but the home learning environment is less likely to improve long-term. Contrariwise, in higher-quality home learning environments, the learning apps may be redundant rather than additive, since core learning processes may already be supported through rich parental engagement.

At the same time, it must be noted that the target group that could benefit most from using the apps (i.e., children from lower-SES families) is underrepresented in this study. Despite efforts to recruit primarily such families, the average SES in the current sample is relatively high, which is likely due to the high cost of living in southern Germany. It is therefore possible that digital compensation is more likely to occur in samples with a lower average SES or among at-risk groups (e.g., Adam et al., 2025; Arnold et al., 2021).

More fundamentally, even well-designed learning apps, while providing practice opportunities and immediate feedback, may lack the dynamic, socially embedded learning processes that characterize most activities within the home learning environment (Wirth et al., 2024). The social and individually adapted aspects of joint activities play a crucial role in facilitating skill development (Vygotsky, 1978), which may lack in the learning apps developed for the current study.

Consequently, although the current study is able to determine precise usage patterns with its focus on the quantitative aspects of app usage, i.e., the exact logging of each second the children used the apps, this approach does not capture the qualitative dimensions of children’s app engagement. It is possible that the quality of app usage plays a more important role than the mere amount of time children spend using learning apps (Neumann, 2018). In particular, from a theoretical perspective, social interactions are crucial for children’s cognitive development (Hirsh-Pasek et al., 2015; Vygotsky, 1978): Learning processes are often most effective when they occur within socially guided interactions in which adults provide scaffolding, feedback, and explanations that are tailored to the child’s current level of understanding. Consequently, simply increasing the quantity of app exposure may not be sufficient to foster learning if children engage with the apps largely independently and without supportive interactions (instead, it might even be detrimental; see, e.g., Adelantado-Renau et al., 2019).

Future research should therefore place greater emphasis on the qualitative and social dimensions of app usage. This includes examining the role of parental involvement and scaffolding during app-based learning, such as guiding children’s attention to relevant content, or linking app activities to everyday experiences (see Neumann, 2018). At the same time, it is important to explore whether and how digital features, such as socially responsive agents within the app, can partially compensate for the absence of adult mediation. Such features would be more adapted to constituting a more knowledgeable other within the techno-subsystem (Johnson & Puplampu, 2008; Vygotsky, 1978) than the more static, less adaptive app format tested in the current study. Furthermore, it could be particularly beneficial for children whose parents are less able to provide frequent scaffolding, for example due to time constraints, language barriers, or limited subject-specific knowledge. Investigating these qualitative aspects of children’s and parents’ app engagement may therefore provide a more nuanced understanding of when and for whom learning apps can effectively support early literacy and numeracy development.

Overall, it seems that child and family characteristics are crucial for skill development, and that neither the home learning environment nor learning apps can fully compensate for these factors when accounting for them. For numeracy skill gains, intelligence emerged as a significant predictor. Consequently, numeracy development may rely to a greater extent on domain-general cognitive abilities such as logical reasoning, working memory, and problem-solving processes (Kapteijns et al., 2025; Peng et al., 2019). In contrast to early literacy development, which is strongly supported through frequent exposure to language and print in everyday interactions (e.g., Dong et al., 2020; Dowdall et al., 2020), numeracy tasks frequently require children to actively manipulate quantities, recognize numerical relations, and apply rule-based reasoning (Raghubar & Barnes, 2017; Stock et al., 2009). Individual differences in cognitive development may thus play a particularly important role for numeracy skill gains. Future research is needed to investigate what design elements and task specifications are required to best support children in their learning process, and whether different design approaches are beneficial for different learning domains.

Finally, for both literacy and numeracy skill gains, SES proved to be a strong predictor. This relation is well documented in prior research, showing persistent socioeconomic disparities in preschool children’s academic skills (e.g., Coddington et al., 2014; Mues et al., 2021). SES is also closely associated with the quality of the home learning environment (Sirin, 2005), a pattern that was reflected in our data as well: greater SES was correlated with higher-quality HLE and HNE. From a socio-ecological perspective, SES shapes the quality of proximal processes, such as parental interaction and, per Johnson and Puplampu (2008), digitally mediated interaction, which support skill development. In this sense, SES can be understood not only as an economic indicator but also as a reflection of differences in cultural and social capital and available resources within the family context (Bradley & Corwyn, 2002).

While the intervention used in this study primarily aimed to promote children’s skills, future interventions should not overlook the important role of parents. By strengthening parents’ knowledge, confidence, and strategies for supporting learning at home, intervention approaches may have more sustainable and far-reaching effects. Consequently, future interventions should place a stronger emphasis on supporting parents, especially those from lower-SES backgrounds (e.g., de Bondt et al., 2020). This could include more widespread parental training programs, for example, through care centers that provide practical strategies for integrating literacy and numeracy learning into everyday family routines. At the same time, further qualitative research and observations of parent–child interactions are needed to better understand how families actually use learning apps in everyday life, including parents’ motivations, expectations, and potential barriers. Such insights could inform the design of apps that are more closely aligned with families’ needs.

Importantly, learning apps often serve a practical function for parents by providing children with independent activities and thereby temporarily relieving parental demands. Future app designs should therefore aim to maintain this supportive role while at the same time offering more targeted learning support. For instance, adaptive features or AI-based guidance could help tailor content and scaffolding to children’s individual learning levels and potentially provide prompts that encourage meaningful engagement. If designed carefully, such technologies might help make digital learning tools more responsive to diverse family contexts and, in the long term, contribute to reducing educational disparities.

### 4.4. Limitations

Despite its strengths, this study has several limitations. First, the HNE was assessed with fewer items than the HLE (12 vs. 24 items). This less comprehensive measurement may have limited the ability to capture the full range and variability of numeracy-related activities within the home. As a consequence, important aspects of the HNE may not have been sufficiently represented. The smaller number of items may also have reduced measurement precision, attenuating associations between HNE and children’s numeracy skill gains.

A second limitation concerns the focus on immediate intervention effects, which allows no statements about potential long-term effects of the learning apps for children’s outcomes. It is possible that the benefits of our apps or the home learning environment emerge only over longer time spans, for example as children repeatedly apply acquired skills in new contexts. Longitudinal follow-up studies would therefore be valuable to determine whether such interventions may produce delayed or cumulative effects on children’s literacy and numeracy development.

Third, we did not control for whether children actually enjoyed or were interested in the apps. This may partly explain differences in app usage times. Although previous findings suggest that children generally liked our apps (Wirth et al., 2024), we were unable to consider interest in the present study. Similarly, our data reflect only the quantitative benefits of the apps, but not the quality of children’s engagement with them. Accordingly, our conclusions regarding the actual mediation of learning processes by digital devices (cf. Vygotsky, 1978) are limited. Investigating the extent to which children actively engage with learning apps (cf. Hirsh-Pasek et al., 2015) and whether they receive parental support during app usage can strengthen future studies and provide more insight into the mechanisms of potential digital compensation.

Fourth, given the sample size of the intervention groups (*n* = 151) and the complexity of the moderation models, our analyses were sensitive only to interaction effects of at least f^2^ = 0.096 (power = 0.90). The absence of a statistically significant HLE/HNE × app usage interaction should therefore be interpreted with caution: it may reflect a genuine absence of compensatory effects, but it may equally result from insufficient power to detect small interaction effects, which are common in field settings. Replication with larger samples is needed before firm conclusions about digital compensation can be drawn.

Fifth, the representativeness of our sample is limited due to its relatively high SES. Although SES showed some variability in our sample, the overall level was high compared to other regions, possibly reflecting the high cost of living in the study area. This should be taken into account when comparing our findings to other studies.

Finally, both the HLE and HNE were only measured at T1. As follow-up measurement for these variables have not been considered, we cannot account for changes in those variables due to and during the intervention. Receiving the apps may have influenced parental behavior, for example, either causing parents to disengage by relying on the apps or prompting greater engagement around the new content. Therefore, the argument that the apps failed to compensate cannot be isolated from the possibility that the intervention itself altered the HLE and HNE.

## 5. Conclusions

This study shows that learning apps can support young children’s gains in literacy and numeracy skills, particularly in comparison to a control group without any additional digital input. However, constrained by limitations in statistical power and a high sample SES, the current study found limited evidence for compensatory effects of learning apps in the context of a low-quality home learning environment. The results point instead to the supplemental potential of learning apps, benefitting children regardless of their prior advantages or disadvantages. Key aspects of both digital and analog home learning environments need to be further investigated in order to determine where digital supplementation is effective and where traditional, analog interventions are more appropriate and potentially enough.

## Figures and Tables

**Table 1:** Descriptive statistics and reliability measures of the whole sample.

Variable	*N*	*M*	*SD*	min	max	Cronbach’s α/McDonald’s Ω
Literacy skills T1	482	0.00	0.47	−1.29	1.14	0.93/0.93
Numeracy skillsT1	472	0.01	0.50	−0.93	2.00	0.94/0.94
Literacy skills T2	486	0.01	0.46	−1.50	0.87	0.91/0.92
Numeracy skills T2	474	0.01	0.52	−1.26	1.25	0.94/0.95
HLE	437	2.46	0.49	0.08	3.74	0.83/0.83
HNE	485	2.61	0.68	0.25	4.00	0.81/0.81
LAUT	499	162.01	374.57	0.00	1969.18	0.95/0.95
LAUT_sq	499	166,266.06	517,549.58	0.00	3,877,669.87	
NAUT	496	219.07	495.92	0.00	2908.52	0.98/0.98
NAUT_sq	497	322,998.84	1,124,907.58	0.00	14,990,525.50	
Sex	500	0.5				
Age	500	60.96	4.61	51.00	75.00	
Intelligence	492	52.2	5.2	12	57	
SES	500	−0.01	0.82	−2.89	1.24	0.75/0.75
MB	486	0.4				
PST	492	2.9	0.9	0.00	4.00	

HLE = home literacy environment at T1. HNE = home numeracy environment at T1. LAUT = literacy-app usage time in minutes. LAUT_sq = LAUT squared. NAUT = numeracy-app usage time in minutes. NAUT_sq = NAUT squared. Sex: 0 = boys, 1 = girls. Age in months. Intelligence assessed at T2; split-half reliability in German contexts between 0.92 and 0.96 (Esser, 2002). SES = socioeconomic status (z-standardized). MB = migration background measured by language mainly spoken at home (0 = German = without MB, 1 = another language than German = with MB). PST = passive screen time.

**Table 2:** Correlation analyses.

		1	2	3	4	5	6	7	8	9	10	11	12	13	14	15
1	Literacy skills T1	−														
2	Numeracy skills T1	0.68 ***	−													
3	Literacy skills T2	0.84 ***	0.66 ***	−												
4	Numeracy skills T2	0.62 ***	0.84 ***	0.67 ***	−											
5	HLE	0.29 ***	0.15 **	0.29 ***	0.13 **	−										
6	HNE	0.24 ***	0.20 ***	0.21 ***	0.21 ***	0.65 ***	−									
7	LAUT	−0.01	0.00	0.12 **	0.00	0.04	0.08	−								
8	LAUT_sq	−0.03	−0.01	0.05	−0.01	0.01	0.06	0.94 ***	−							
9	NAUT	−0.04	−0.01	−0.10 *	0.05	−0.05	−0.02	−0.18 ***	−0.14 **	−						
10	NAUT_sq	−0.07	−0.02	−0.11 *	0.01	−0.06	−0.03	−0.12 **	−0.09 *	0.89 ***	−					
11	Sex	0.09	−0.09 *	0.09	−0.11 *	0.05	−0.03	−0.06	−0.08	−0.01	−0.01	−				
12	Age	0.13 *	0.25 ***	0.15 ***	0.26 ***	−0.16 ***	−0.06	0.02	0.00	0.02	0.02	−0.05	−			
13	Intelligence	0.40 ***	0.35 ***	0.42 ***	0.38 ***	0.25 ***	0.22 **	−0.03	−0.03	−0.03	−0.06	0.10 *	0.09	−		
14	SES	0.42 ***	0.27 ***	0.41 ***	0.27 ***	0.29 ***	0.10 *	−0.07	−0.12 **	−0.04	−0.09	−0.01	−0.21 ***	0.24 ***	−	
15	MB	−0.39 ***	−0.22 ***	−0.38 ***	−0.17 ***	−0.16 ***	0.00	0.03	0.06	0.03	0.06	−0.02	0.01	−0.18 ***	−0.30 ***	−
16	PST	0.09 *	0.01	0.09	−0.03	0.09*	0.01	0.00	−0.01	−0.01	0.03	−0.08	−0.06	−0.05	0.12 **	−0.09

HLE = home literacy environment at T1. HNE = home numeracy environment at T1. LAUT = literacy-app usage time in minutes. LAUT_sq = LAUT squared. NAUT = numeracy-app usage time in minutes. NAUT_sq = NAUT squared. Sex: 0 = boys, 1 = girls. Age in months. Intelligence assessed at T2. SES = socioeconomic status (z-standardized). MB = migration background measured by language mainly spoken at home (0 = German = without MB, 1 = another language than German = with MB). PST = passive screen time. * *p* < 0.05. ** *p* < 0.01. *** *p* < 0.001.

## Data Availability

The raw data supporting the conclusions of this manuscript as well as the coding script can be found under https://osf.io/e2d74/overview?view_only=810b35145e0d4069aed9ae48bb58f16e (accessed on 29 July 2026).

## Supplementary Materials

The following supporting information can be downloaded at: https://www.mdpi.com/article/10.3390/bs16081304/s1, Table S1: Descriptive statistics within the literacy group (*n* = 151); Table S2: Descriptive statistics within the numeracy group (*n* = 151); Table S3: Literacy learning apps; Table S4: Numeracy learning apps; Table S5: Pooled regression models on differences in literacy/numeracy skill gains depending on intervention group membership (H1a, H1b); Table S6: Pooled regression models on literacy/numeracy skill gains predicted by literacy/numeracy app usage time (H2a, H2b); Table S7: Pooled regression models within the literacy group (*n* = 151) to assess interaction between home literacy environment and apps on literacy gains (H3a); Table S8: Model fit indices of multiple regression models (H3a, H3b); Table S9: Pooled regression models within the numeracy group (*n* = 151) to assess interaction between home numeracy environment and apps on numeracy gains (H3b); Figure S1: Quadratic effects of the relation between app usage and skills at T2 for literacy (left) and numeracy (right); Supplementary S1: Parental survey: Home literacy and home numeracy environment at T1.

## Abbreviations

The following abbreviations are used in this manuscript:

HLE	Home Literacy Environment
HNE	Home Numeracy Environment
LAUT	Literacy app usage time
LAUT_sq	Squared literacy app usage time
NAUT	Numeracy app usage time
NAUT_sq	Squared numeracy app usage time
SES	Socioeconomic status
PST	Passive screen time
MB	Migration background
