# Supplementary material for: Digital Compensation? Investigating the Moderating Role of the Home Learning Environment in the Effectiveness of Learning Apps
Source: Behav Sci (Basel). 2026 Jul 31;16(8):1304. doi: 10.3390/bs16081304 (PMC13509475; doi:10.3390/bs16081304)
Supplement: Supplementary file 1 [file behavsci-16-01304-s001.zip › behavsci-4288346-supplementary.pdf]

Supplementary Materials

Table of Contents

Table S1. *Descriptive statistics within the literacy group (n = 151)*.......... 2

Table S2. *Descriptive statistics within the numeracy group (n = 151)*.......... 3

Table S3. *Literacy learning apps.*.......... 4

Table S4. *Numeracy learning apps.*.......... 9

Table S5. *Pooled regression models on differences in literacy/numeracy skill gains depending on intervention group membership (H1a, H1b).*.......... 15

Table S6. *Pooled regression models on literacy/numeracy skill gains predicted by literacy/numeracy app usage time (H2a, H2b).*.......... 17

Table S7. *Pooled regression models within the literacy group (n = 151) to assess interaction between home literacy environment and apps on literacy gains (H3a).*.......... 19

Table S8. *Model fit indices of multiple regression models (H3a, H3b).*.......... 21

Table S9. *Pooled regression models within the numeracy group (n = 151) to assess interaction between home numeracy environment and apps on numeracy gains (H3b).*.......... 22

Figure S1. *Quadratic effects of the relation between app usage and skills at T2 for literacy (left) and numeracy (right).*.......... 24

Supplementary S1. *Parental survey: Home literacy and home numeracy environment at T1*.......... 26

Table S1. *Descriptive statistics within the literacy group (n = 151).*

Variable	*N*	*M*	*SD*	min	max
Literacy skills T1	146	-0.01	0.48	-1.16	0.97
Numeracy skills T1	140	0.02	0.52	-0.93	2.00
Literacy skills T2	147	0.11	0.44	-1.33	0.80
Numeracy skills T2	145	-0.00	0.52	-1.09	1.25
HLE	129	2.55	0.42	1.54	3.74
HNE	148	2.70	0.65	0.50	3.92
LAUT	150	538.94	514.212	0.00	1969.18
LAUT_sq	150	553111.74	824530.52	0.00	3877669.87
Sex	151	0.50			
Age	151	60.78	4.43	51.00	73.00
Intelligence	147	52.01	5.77	19	57
SES	151	0.04	0.79	-2.73	1.24
MB	147	0.37			
PST	147	2.86	0.91	0.00	4.00

HLE = home literacy environment at T1. HNE = home numeracy environment at T1. LAUT = literacy-app usage time in minutes. LAUT_sq = LAUT squared. Sex: 0 = boys, 1 = girls. Age in months. Intelligence assessed at T2. SES = socioeconomic status (z-standardized). MB = migration background measured by language mainly spoken at home (0 = German = without MB, 1 = another language than German = with MB). PST = passive screen time. Literacy and numeracy skills are z-standardized.

Table S2. *Descriptive statistics within the numeracy group (n = 151).*

Variable	*N*	*M*	*SD*	min	max
Literacy skills T1	146	-0.00	0.50	-1.29	1.08
Numeracy skills T1	145	-0.02	0.51	-0.93	1.88
Literacy skills T2	147	-0.04	0.49	-1.44	0.85
Numeracy skills T2	148	0.04	0.51	-1.26	1.23
HLE	134	2.44	0.49	0.56	3.58
HNE	145	2.64	0.70	0.58	4.00
NAUT	148	757.31	716.52	0.00	3871.76
NAUT_sq	148	1083454.29	1856866.66	0.00	14990525.50
Sex	151	0.53	0.50	0	1
Age	151	60.99	4.51	53.00	75.00
Intelligence	150	52.35	4.34	30	57
SES	151	0.00	0.85	-2.89	1.24
MB	148	0.43	0.50	0	1
PST	149	2.81	0.95	0.00	4.00

HLE = home literacy environment at T1. HNE = home numeracy environment at T1. NAUT = numeracy-app usage time in minutes. NAUT_sq = NAUT squared. Sex: 0 = boys, 1 = girls. Age in months. Intelligence assessed at T2. SES = socioeconomic status (z-standardized). MB = migration background measured by language mainly spoken at home (0 = German = without MB, 1 = another language than German = with MB). PST = passive screen time. Literacy and numeracy skills are z-standardized.

Table S3. *Literacy learning apps.*

App Name	Month	Time played in minutes total<br>Max; *M(SD)*	Description	Screenshot	Literacy Skills
Memory	1	366.97;<br>71.90(78.64)	This app corresponds to the traditional analogue memory game. All cards exist in pairs and are placed face down. Players turn the cards over by tapping them and memorize the letters on them. The aim of the game is to uncover two matching cards directly after each other (e.g., “A” and “A”).	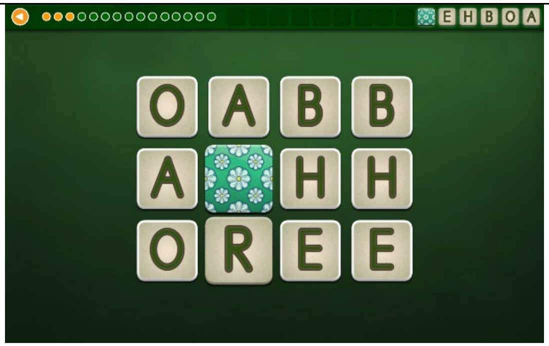	Active and passive letter knowledge
Letter drawing	1	361.93;<br>61.22(59.14)	In this app, letters are to be traced with the finger using displayed lines. Players follow a displayed arrow that guides them tracing letters.	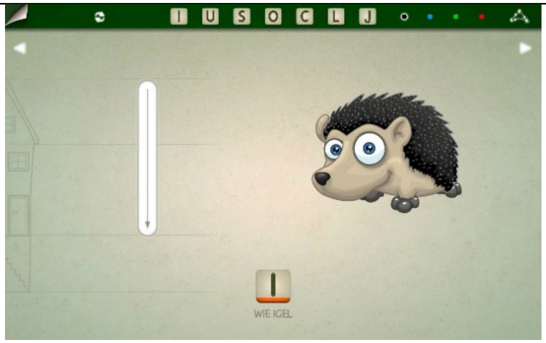	Active and passive letter knowledge

App Name	Month	Time played in minutes total	Description	Screenshot	Literacy Skills
Painting with letters	1	835.85;<br>142.48(133.94)	In this app, children color pictures. Both the areas of the picture and the colors are marked with letters. E.g., an area marked with "A" is to be colored with the color marked with "A" (e.g., blue).	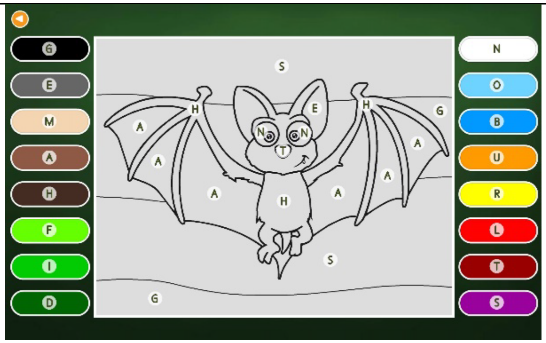	Active and passive letter knowledge
Letter sorting	1	394.23;<br>62.16(66.34)	In this app, children sort a series of letters (e.g., D-A-B-C) in the correct order according to the alphabet (e.g., A-B-C-D). Pictures of animals with the corresponding initial letter simplify the matching.	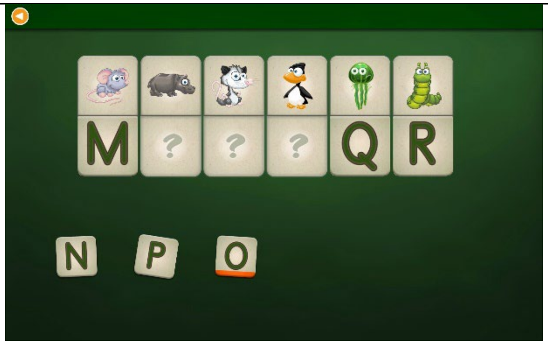	Active and passive letter knowledge
Initial letter sounds	2	681.77;<br>67.38(87.98)	In this app, there are different levels for each letter (e.g., level "L"). Children are presented with animals one after the other (e.g., cow, lynx, lion, whale). Animals with the same initial letter as the letter of the level should be selected (e.g., lynx, lion).	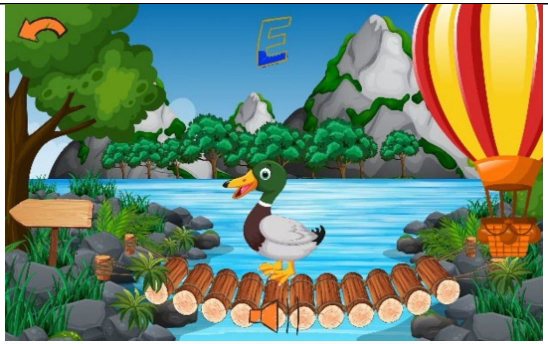	Phonological awareness

App Name	Month	Time played in minutes total	Description	Screenshot	Literacy Skills
Snakes & Letters	2	271.90; 72.63(77.44)	This app corresponds to the traditional Snakes & Ladders game. The board and the dice are marked with letters. This game can be played in multi-player mode.	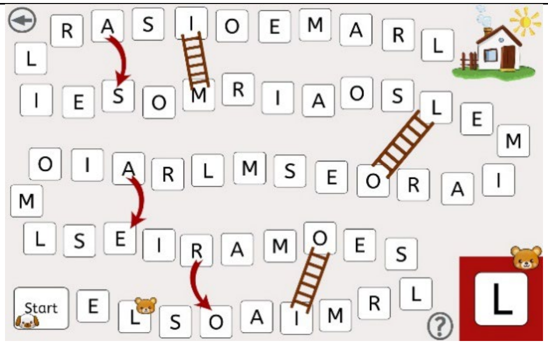	Active and passive letter knowledge
Find the vowels	3	357.93; 23.48(42.46)	In this app, a letter is presented visually and audibly to the children (e.g., “A”). From two, three, or four objects, children are now to select the appropriate one that incorporates the letter (e.g., “panda”).	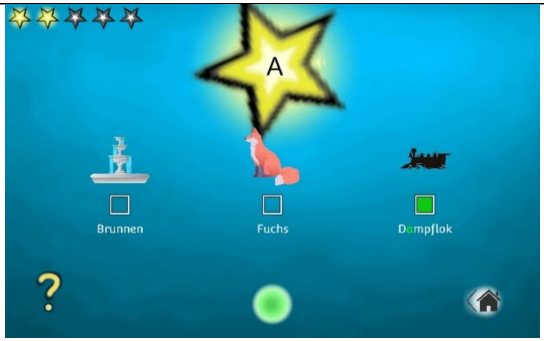	Active and passive letter knowledge
Finding pairs	3	160.87; 23.31(33.09)	In this app, children are visually and audibly presented with the image of an object (e.g., “Wind”). They must now select from four other images, whose pronunciation they can listen to, the image that rhymes with the presented object (e.g., “Kind”).	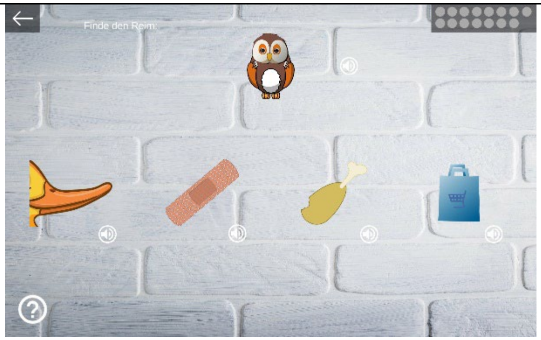	Phonological awareness

App Name	Month	Time played in minutes total	Description	Screenshot	Literacy Skills
Sentence comprehension	4	151.52;<br>28.12(40.64)	In this app, children are read a short story. After each paragraph, they are asked several questions about the content of the story, which they can answer by selecting the correct image out of four (e.g., "Do you remember what season it was?" using images depicting the four seasons).	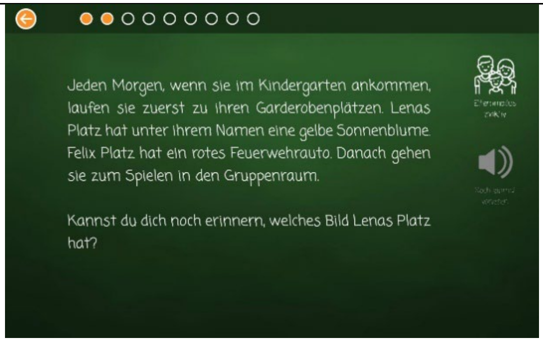 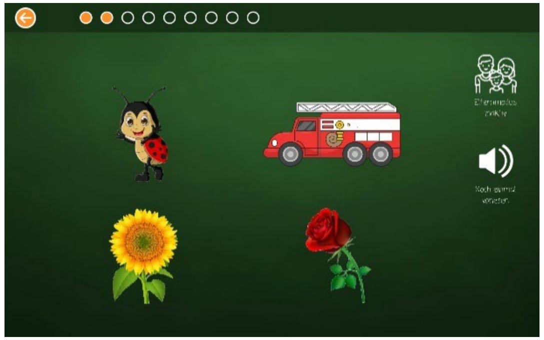	Passive vocabulary; early literacy knowledge
Letter-Domino	4	174.58;<br>20.43(34.21)	In this app, the task is to place game pieces together like in a traditional analogue domino game. The game pieces have letters and animal faces on them. The animals should match with their initial letters.	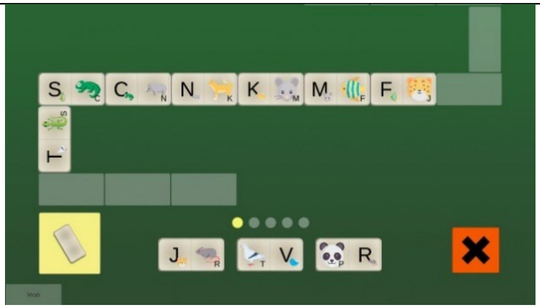	Active and passive letter knowledge

App Name	Month	Time played in minutes total	Description	Screenshot	Literacy Skills
Magic potion	5	111.37;<br>18.26(25.86)	In this app, children help a witch brew a magic potion. For this, they need various ingredients. The witch spells out in sounds which object is needed next (e.g., P-O-T). The children must select the correct one from three given objects (e.g., the pot).	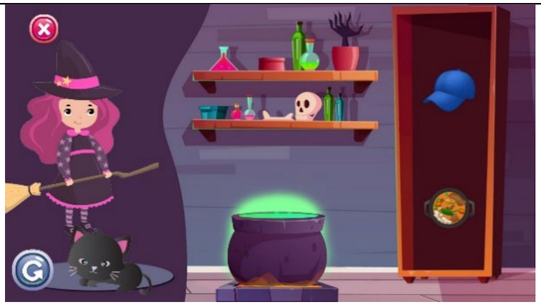	Phonological awareness
Word puzzle by PixaLink[1]	5	290.62;<br>15.54(45.00)	In this app, predetermined letters must be connected in the correct order to find the corresponding word for the visually depicted search object.	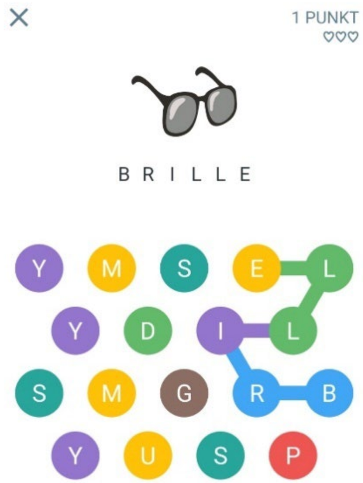	Early literacy knowledge

[1]The word puzzle app was not designed and developed within the framework of the Learning4Kids study.

Table S4. *Numeracy learning apps.*

App Name	Month	Time played in minutes total<br>Max; *M(SD)*	Description – Numeracy Apps	Screenshot	Trained Skills
Memory (Numbers)	1	815.63;<br>64.98(102.65)	This app corresponds with the traditional analogue memory game. All cards exist in pairs and are placed face down. Children turn the cards over by tapping and memorize the numbers on them. The aim of the game is to uncover two matching cards directly after each other. Number names are provided verbally when tapping in the easier levels.	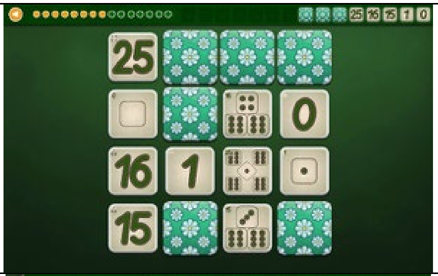	Number symbol knowledge, knowledge of numeric representation
Number drawing	1	617.57;<br>70.99(82.31)	In this app, the aim is to trace the lines with your finger following displayed lines. Children follow a displayed arrow and then trace whole number symbols. Number names are provided verbally for each item.	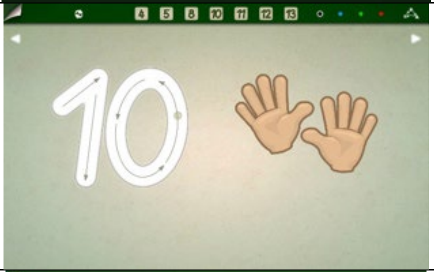	Number symbol knowledge, knowledge of numeric representation
Painting with numbers	1	818.05;<br>170.48(153.85)	In this app, children colour pictures. Both the areas of the picture and the colours are marked with numbers. The colours to be used for certain areas are linked and displayed using a legend with numbers. Number names are provided verbally when tapping.	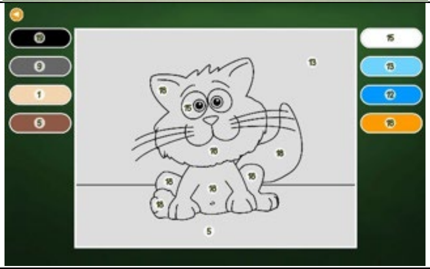	Number symbol knowledge

App Name	Month	Time played in minutes total	Description – Numeracy Apps	Screenshot	Trained Skills
Number sorting	1	808.37; 66.13(89.76)	In this app, children sort a series of numbers in the correct order. The children's task is to correctly assign the numbers, which are offered below in a randomized order and in different forms of representation. Number names are provided verbally.	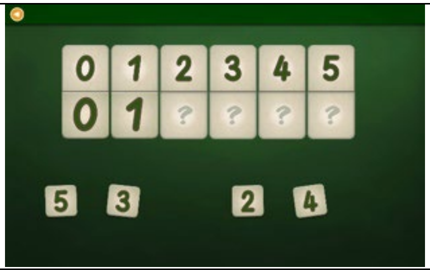	Number symbol knowledge, Ordinal number knowledge,
Build a number rocket	1	699.68; 66.65(77.65)	In this app, children build a number rocket. On the right-hand side they see a rocket with empty fields and on the left side a selection of rocket components with different numbers. The rocket must be constructed with equal/ascending/descending number sequences. Number names are provided verbally.	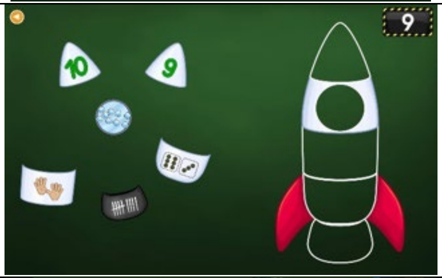	Number symbol knowledge, knowledge of numeric representations, ordinal number knowledge
Collecting nuts (numbers)	2	304.60; 38.20(50.15)	In this app, a squirrel runs along a predetermined path to a destination. The children are provided with the information how many (different) nuts the squirrel needs and they are asked to tap the number of nuts accordingly.	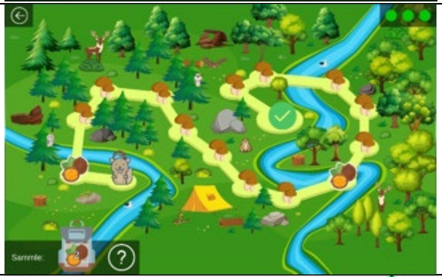	Counting, inclusion and relations, number sequence forwards, cardinal number knowledge
Snakes & Ladders (Numbers)	2	1124.93; 116.65(176.18)	This app corresponds to the traditional Snakes & Ladders game. The board and the dice are marked with numbers. This game can be played in multi-player mode. Number names are provided verbally.	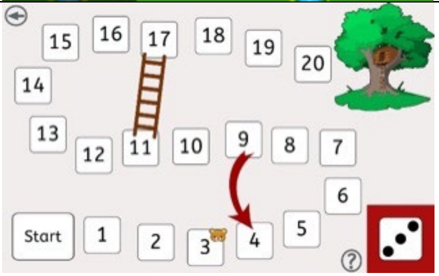	Number symbol knowledge, number sequence forwards, number sequence backwards, number predecessors and successors

App Name	Month	Time played in minutes total	Description – Numeracy Apps	Screenshot	Trained Skills
Tap it! Numbers	2	491.95; 32.09(54.18)	In this app, children should select numbers by tapping them as quickly as possible. The children are instructed to find and tap the target number "x". The children are asked to tap the indicated number "x" and not the 1-3 distractor numbers that also appear regularly. After tapping (or when the numbers were not tapped on fast enough), the numbers disappear. In a second game phase, children are asked to count.	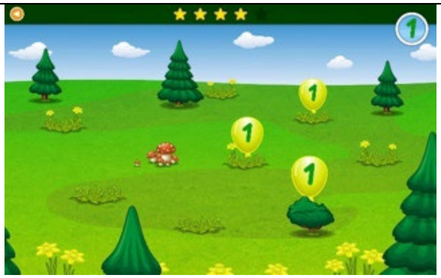	Number symbol knowledge; knowledge of numeric representations, number sequence forwards, number sequence backwards
Mathe-marmite[1]	2	1449.37; 112.20(187.93)	In this app, children create monsters by using their counting abilities to put the correct number of specific ingredients into a magic pot. The game mainly contains counting tasks, but different representations for the numbers.	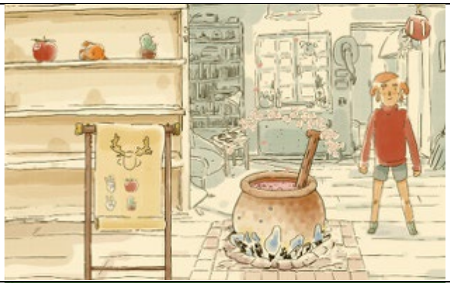	Number symbol knowledge, counting, knowledge of numeric representations
Measurement app	3	556.82; 61.66(89.66)	In this app, children see a ruler in the middle of the screen. Objects and animals of different sizes appear next to the ruler. There is a number box under the ruler and the children should measure the correct size and choose the corresponding number card. In a later level, they also should build the size of the objects/animals with building blocks.	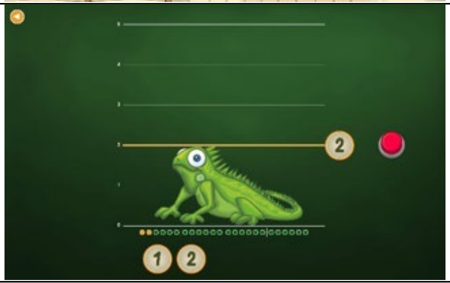	Number symbol knowledge, inclusion and relations, comparison and measurement, easy calculations

App Name	Month	Time played in minutes total	Description – Numeracy Apps	Screenshot	Trained Skills
Finding pairs (Numbers)	3	167.77; 17.37(27.90)	In this app, children are asked to choose the appropriate object out of two to four objects, that corresponds to a given number. The numbers are provided verbally.	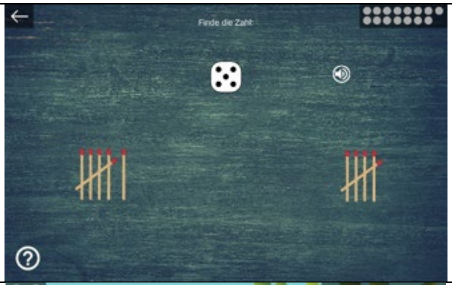	Number symbol knowledge; Knowledge of numeric representations
Count and compare	3	334.58; 24.21(46.20)	In this app, children have to count the number of animals correctly and choose the correct number card Later, they have two identify the correct number of animals according to a given number symbol that is also named verbally. In the final levels, the quantities need to be compared (larger, smaller, equal).	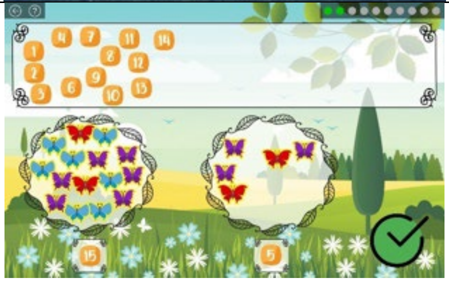	Number symbol knowledge, ordinal number bars, cardinality, inclusion and relations, counting, number sequence forwards, number sequence backwards,
Counting the balloons	4	224.95; 12.5(26.64)	In this app, children are asked to count balloons and select the correct number of balloons by tapping. in later and more difficult levels, children are asked to do easy calculations with the balloons.	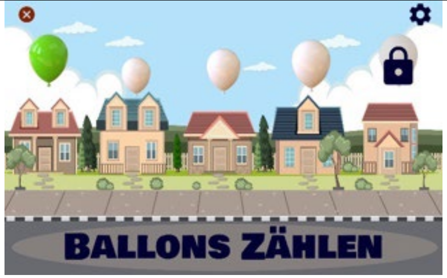	Number symbol knowledge, counting, number sequence forwards, number sequence backwards, addition & subtraction

App Name	Month	Time played in minutes total	Description – Numeracy Apps	Screenshot	Trained Skills
Number-Domino	4	434.25; 30.62(58.68)	In this app, children need to place the game pieces together like in a classic analogue domino game and solve the domino game correctly. The numbers on the domino tokens differ in size and representation.	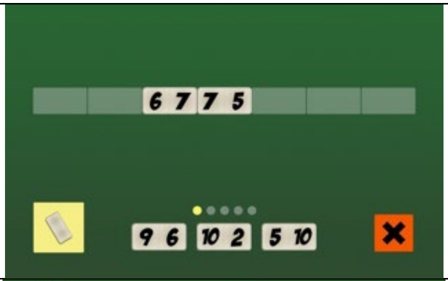	Number symbol knowledge, knowledge of numeric representations
Connect the number dots	4	213.93; 17.95(32.44)	In this app, the task is to paint or complete a picture by connecting dots to form lines. The points to be connected are displayed with numbers. In later levels the numbers go up to 20 and should be connected backwards.	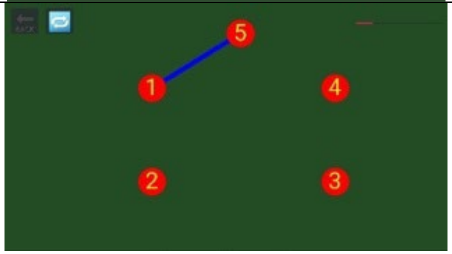	Number symbol knowledge, knowledge of numeric representations, counting, number sequence forwards, number sequence backwards
Learn the Clock	5	276.27; 14.86(34.78)	With this app, children learn the clock. First, times are displayed with whole hours. Later, children have to identify the time on the clock with smaller units. Finally, they have to conduct easy calculations with time periods.	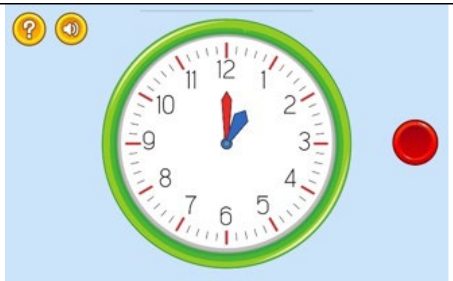	Number symbol knowledge, reading the clock, calculation with time.

App Name	Month	Time played in minutes total	Description – Numeracy Apps	Screenshot	Trained Skills
Count and sort objects	5	153.03; 11.06(22.15)	In this app, children have to count and assign different objects and number symbols to the corresponding number symbol on a box.	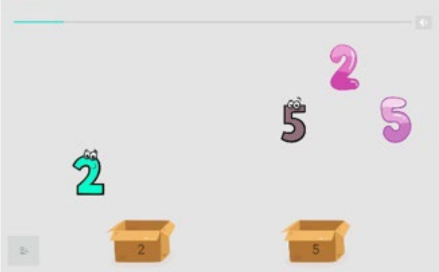	Number symbol knowledge, knowledge of numeric representations,
Categorize numbers	5	772.42; 24.76(80.23)	In this app, children are supposed to find the identical objects and put them together in a row. Next, they have to count them and assign the correct number.	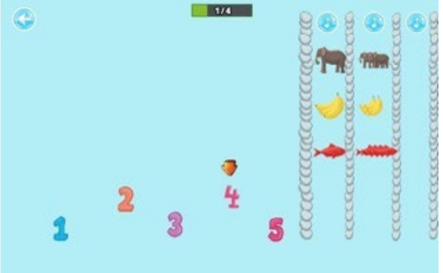	Number symbol knowledge, inclusion, counting

[1]The app Mathemarmite was not designed and developed within the framework of the Learning4Kids study.

Table S5. *Pooled regression models on differences in literacy/numeracy skill gains depending on intervention group membership (H1a, H1b).*

	Variable	Std. ß	*SE*	*t(df)*	*p*	R^2
Model A1						.73
	Intercept (Literacy app group)	.23	.04	5.92(478.18)	< .001	
	Literacy skills T1	.83	.02	35.24(467.83)	< .001	
	Numeracy app group	-.36	.06	-5.85(471.21)	< .001	
	Tablet-control group	-.35	.07	-5.01(460.73)	< .001	
	Control group	-.33	.07	-4.89(474.04)	< .001	
Model A2						.75
	Intercept (Literacy app group)	.24	.09	2.55(438.43)	.011	
	Literacy skills T1	.73	.03	24.50(446.81)	< .001	
	Numeracy app group	-.36	.06	-5.96(450.08)	< .001	
	Tablet-control group	-.36	.07	-4.97(436.17)	< .001	
	Control group	-.37	.07	-5.41(452.88)	< .001	
	Sex	.06	.05	1.22(453.50)	.225	
	Age	.07	.03	2.68(440.58)	.008	
	Intelligence	.10	.03	3.56(390.66)	< .001	
	SES	.07	.03	2.67(448.13)	.008	
	MB	-.11	.05	-1.96(451.72)	.050	
	PST	.01	.03	0.49(427.78)	.624	
Model B1						.71
	Intercept (Numeracy app group)	.10	.04	2.15(474.39)	.032	
	Numeracy skills T1	.85	.02	34.35(469.38)	< .001	
	Literacy app group	-.12	.06	-1.96(458.26)	.051	
	Tablet-control app group	-.14	.07	-1.88(453.42)	.060	
	Control group	-.19	.07	-2.68(447.83)	.008	
Model B2						.73
	Intercept (Numeracy app group)	.21	.10	2.21(453.85)	.028	
	Numeracy skills T1	.79	.03	27.44(449.15)	< .001	
	Literacy app group	-.10	.06	-1.56(439.92)	.119	
	Tablet-control app group	-.13	.07	-1.84(434.29)	.066	

Variable	Std. ß	*SE*	*t(df)*	*p*	R^2
Control group	-.17	.07	-2.40(421.38)	.017	
Sex	-.09	.05	-1.81(433.97)	.072	
Age	.07	.03	2.63(421.34)	.009	
Intelligence	.09	.03	3.40(444.94)	.001	
SES	.07	.03	2.46(440.54)	.014	
MB	.06	.05	1.02(428.57)	.310	
PST	-.04	.03	-1.29(439.68)	.199	

Sex: 0 = boys, 1 = girls. Intelligence assessed at T2. SES = socioeconomic status (z-standardized). MB = migration background measured by language mainly spoken at home (0 = German = without MB, 1 = another language than German = with MB). PST = passive screen time.

Table S6. *Pooled regression models on literacy/numeracy skill gains predicted by literacy/numeracy app usage time (H2a, H2b).*

	Variable	Std. ß	*SE*	*t(df)*	*p*	R^2
Model A1						.67
	Intercept	.20	.06	3.50(139.06)	< .001	
	Literacy skills T1	.73	.04	16.47(142.98)	< .001	
	LAUT	.34	.10	3.50(140.48)	< .001	
	LAUT_sq	-.00	.00	-3.30(140.69)	.001	
Model A2						.69
	Intercept	.28	.18	1.61(127.71)	.110	
	Literacy skills T1	.62	.06	10.66(131.61)	< .001	
	LAUT	.29	.10	3.05(130.80)	.003	
	LAUT_sq	.00	.00	-2.50(131.68)	.014	
	Sex	.02	.09	0.24(131.30)	.814	
	Age	.03	.05	0.54(131.83)	.593	
	Intelligence	.06	.05	1.39(130.37)	.168	
	SES	.17	.06	3.07(133.33)	.003	
	MB	-.04	.11	-0.35(130.38)	.724	
	PST	-.04	.05	-0.74(127.87)	.460	
Model B1						.69
	Intercept	.02	.06	0.26(129.82)	.797	
	Numeracy skills T1	.79	.05	17.58(141.14)	< .001	
	NAUT	.21	.07	3.03(111.43)	.003	
	NAUT_sq	.00	.00	-2.66(115.98)	.009	
Model B2						.75
	Intercept	.13	.16	0.421(129.09)	.421	
	Numeracy skills T1	.68	.05	13.81(130.68)	< .001	
	NAUT	.18	.06	2.86(110.02)	.005	
	NAUT_sq	.00	.00	-2.04(114.23)	.044	
	Sex	-.13	.09	-1.46(127.78)	.148	
	Age	.08	.05	1.64(130.45)	.103	
	Intelligence	.20	.06	3.36(130.29)	.001	

Variable	Std. ß	*SE*	*t(df)*	*p*	R^2
SES	.12	.05	2.49(129.56)	.014	
MB	-.03	.10	-0.26(126.04)	.794	
PST	-.04	.05	-0.89(131.28)	.374	

LAUT = literacy app usage time in minutes. LAUT_sq = LAUT squared. NAUT = numeracy app usage time in minutes. NAUT_sq = NAUT squared. Sex: 0 = boys, 1 = girls. Intelligence assessed at T2. SES = socioeconomic status (z-standardized). MB = migration background measured by language mainly spoken at home (0 = German = without MB, 1 = another language than German = with MB). PST = passive screen time.

Table S7. *Pooled regression models within the literacy group (n = 151) to assess interaction between home literacy environment and apps on literacy gains (H3a).*

	Variable	Std. ß	*SE*	*t(df)*	*p*	R^2
Model A1						.65
	Intercept	.25	.05	5.37(142.53)	< .001	
	Literacy skills T1	.75	.05	16.36(145.05)	< .001	
Model A2						.68
	Intercept	.30	.15	1.99(136.75)	.049	
	Literacy skills T1	.69	.05	14.90(136.12)	< .001	
	LAUT	.31	.09	3.29(134.69)	.001	
	LAUT_sq	-.00	.00	-3.09(134.18)	.002	
	HLE	.12	.05	2.44(111.69)	.016	
	PST	-.04	.05	-0.72(135.78)	.472	
Model A3						.69
	Intercept	.27	.15	1.76(132.89)	.080	
	Literacy skills T1	.68	.05	14.36(131.09)	< .001	
	LAUT	.35	.10	3.61(131.91)	< .001	
	LAUT_sq	-.00	.00	-3.16(130.14)	.002	
	HLE	.16	.06	2.72(113.85)	.008	
	HLExLAUT	-.06	.11	-0.56(114.75)	.576	
	HLExLAUT_sq	-.00	.00	-0.06(122.47)	.952	
	PST	-.03	.05	-0.53(131.95)	.600	
Model A4						.71
	Intercept	.29	.18	1.66(124.39)	.099	
	Literacy skills T1	.60	.06	10.17(128.01)	< .001	
	LAUT	.30	.10	3.01(125.66)	.003	
	LAUT_sq	-.00	.00	-2.42(125.59)	.017	
	HLE	.12	.06	1.97(105.27)	.051	
	HLExLAUT	-.05	.11	-0.43(109.67)	.670	
	HLExLAUT_sq	.00	.00	0.08(117.10)	.940	
	Sex	.00	.09	0.03(126.20)	.978	
	Age	.05	.05	0.89(126.15)	.376	

Variable	Std. ß	*SE*	*t(df)*	*p*	R^2
Intelligence	.05	.05	1.01(125.15)	.312	
SES	.14	.06	2.33(124.82)	.021	
MB	-.04	.11	-0.33(126.05)	.741	
PST	-.04	.05	-0.74(124.52)	.461	

LAUT = literacy-app usage time in minutes. LAUT_sq = LAUT squared. HLE = home literacy environment. Sex: 0 = boys, 1 = girls. Intelligence assessed at T2. SES = socioeconomic status (z-standardized). MB = migration background measured by language mainly spoken at home (0 = German = without MB, 1 = another language than German = with MB). PST = passive screen time.

Table S8. *Model fit indices of multiple regression models (H3a, H3b).*

	Model	Adj. R^2	LL	*df*	AIC	BIC	aBIC
Literacy (H3a)							
	0	.64	-127.85	1	261.70	270.75	258.74
	1	.67	-115.56	5	245.13	266.13	246.33
	2	.67	-113.10	7	244.19	271.23	247.48
	3	.68	-106.78	12	241.56	283.43	250.05
Numeracy (H3b)							
	0	.67	-127.13	1	260.26	269.31	257.30
	1	.69	-120.28	5	254.55	275.63	255.76
	2	.68	-120.00	7	258.00	285.10	261.29
	3	.73	-105.19	12	238.39	280.35	246.88

LL = loglikelihood. AIC = Akaike Information Criterion. BIC = Bayesian Information Criterion. aBIC = sample-size adjusted BIC.

Table S9. *Pooled regression models within the numeracy group (n = 151) to assess interaction between home numeracy environment and apps on numeracy gains (H3b).*

	Variable	Std. ß	*SE*	*t(df)*	*p*	R^2
Model B1						.67
	Intercept	.09	.05	1.94(142.30)	.055	
	Numeracy skills T1	.80	.05	17.36(145.27)	< .001	
Model B2						.70
	Intercept	.10	.15	0.65(134.30)	.518	
	Numeracy skills T1	.78	.05	16.60(137.27)	< .001	
	NAUT	.20	.07	2.97(109.97)	.004	
	NAUT_sq	-.00	.00	-2.56(114.47)	.012	
	HNE	.02	.05	0.38(134.74)	.701	
	PST	-.03	.05	-0.59(134.61)	.559	
Model B3						.70
	Intercept	.09	.15	0.62(132.07)	.535	
	Numeracy skills T1	.78	.05	16.41(135.25)	< .001	
	NAUT	.20	.07	2.91(107.10)	.004	
	NAUT_sq	-.00	.00	-2.46(111.78)	.015	
	HNE	.03	.05	0.49(131.15)	.624	
	HNExNAUT	-.05	.10	-0.57(129.18)	.571	
	HNExNAUT_sq	.00	.00	0.46(132.49)	.648	
	PST	-.03	.05	-0.56(132.72)	.576	
Model B4						.75
	Intercept	.11	.17	0.69(126.27)	.493	
	Numeracy skills T1	.67	.05	13.45(126.77)	< .001	
	NAUT	.18	.06	2.84(108.36)	.005	
	NAUT_sq	-.00	.00	-2.03(113.15)	.045	
	HNE	.00	.05	0.01(128.23)	.988	
	HNExNAUT	.04	.09	0.45(126.28)	.650	
	HNExNAUT_sq	-.00	.00	-0.76(128.19)	.450	
	Sex	-.13	.09	-1.41(124.47)	.161	
	Age	.09	.05	1.78(126.69)	.078	

Variable	Std. ß	*SE*	*t(df)*	*p*	R^2
Intelligence	.22	.06	3.45(126.89)	< .001	
SES	.11	.05	2.21(126.08)	.029	
MB	-.02	.10	-0.18(125.50)	.859	
PST	-.02	.05	-0.39(126.66)	.699	

NAUT = numeracy app usage time in minutes. NAUT_sq = NAUT squared. HNE = home numeracy environment. Sex: 0 = boys, 1 = girls. Intelligence assessed at T2. SES = socioeconomic status (z-standardized). MB = migration background measured by language mainly spoken at home (0 = German = without MB, 1 = another language than German = with MB). PST = passive screen time.

Figure S1. *Quadratic effects of the relation between app usage and skills at T2 for literacy (left) and numeracy (right).*

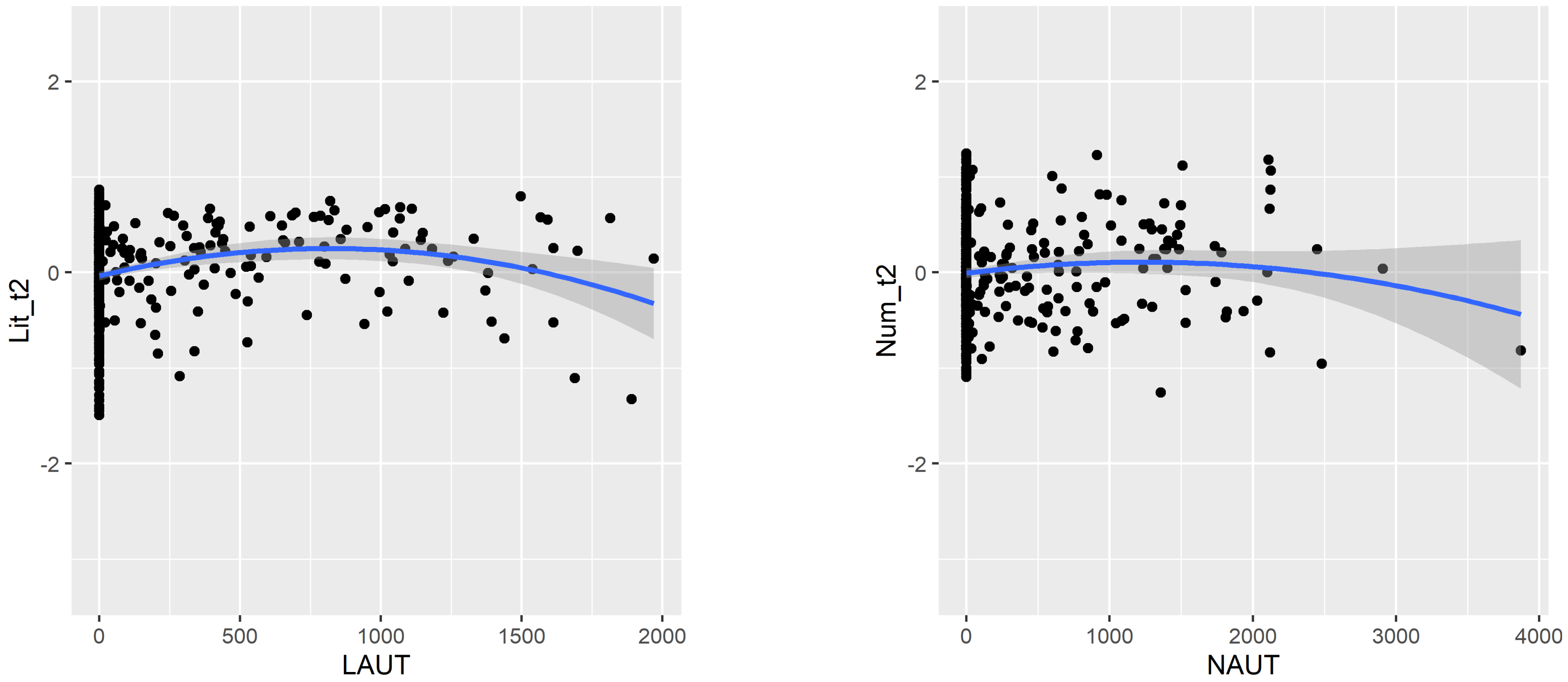

LAUT = literacy app usage time in minutes. NAUT = numeracy app usage time in minutes. Lit_t2 = literacy skills at T2 (z-standardized). Num_T2 = numeracy skills at T2 (z-standardized).

Figure S2. *Model fit indices of multiple regression models for literacy (H3a, left) and numeracy (H3b, right).*

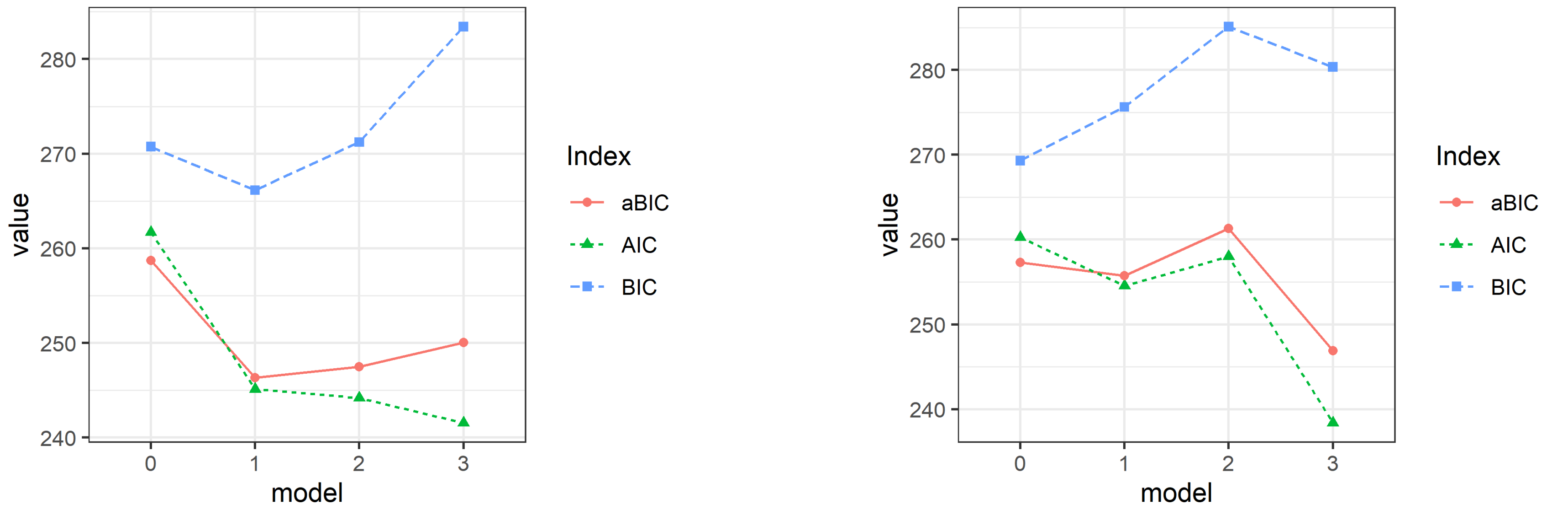

BIC = Bayesian information criterion; AIC = Akaike's information criterion; aBIC = adjusted BIC.

Supplementary S1. *Parental survey: Home literacy and home numeracy environment at T1.*

1) Home Literacy Environment

In case you already read to your child: How often do you and your partner read to your child at the moment? This also includes looking at picture books together, also those with less pictures or made out of other materials like cloth or wood.

☐ several times a day ☐ daily ☐ several times a week ☐ once a week ☐ less often/never

Does your child look at picture books by himself/herself?

☐ several times a day ☐ daily ☐ several times a week ☐ once a week ☐ less often/never

How many books do you approximately own in your household?
(This includes also non-fiction books and cooking books, but no e-books)

☐ none ☐ 1-10 ☐ 11-50 ☐ 51-100 ☐ 100-150 ☐ 151-200 ☐ > than 200 books

How many children’s books or picture and Pixi books do you own?

☐ none ☐ 1-5 ☐ 6-10 ☐ 11-20 ☐ 21-50 ☐ 51-100 ☐ > than 100 books

How often do you visit libraries/ public bookstores with your child at the moment?

☐ several times a week ☐ weekly ☐ approx. once a month ☐ less often ☐ never

How often do you sing with your child or recite poems or rhymes?

☐ several times a day ☐ daily ☐ several times a week ☐ once a week ☐ less often/never

How often do you read yourself, e.g. a printed book, the newspaper or a printed journal?

☐ several times a day ☐ daily ☐ several times a week ☐ once a week ☐ less often/never

And how often do you read these things in digital form, i.e. on the computer, tablet, smartphone or ebook reader?

☐ several times a day ☐ daily ☐ several times a week ☐ once a week ☐ less often/never

How often does your partner read a printed book, newspaper or journal? *(recoded to a 4-0 scale)*
☐ several times a day ☐ daily ☐ several times a week ☐ once a week ☐ less often/never

And how often does he/she read digitally, meaning on the computer, tablet, smartphone or e-reader? *(recoded to a 4-0 scale)*
☐ several times a day ☐ daily ☐ several times a week ☐ once a week ☐ less often/never

On a usual workday: How many hours does your child watch movies, videoclips, series on TV or on PC / laptop / tablet?
☐ more than 3 hours ☐ 2-3 hours ☐ 1-2 hours ☐ 30-60min ☐ less often/never

And how many hours on a usual day on the weekend?
☐ more than 3 hours ☐ 2-3 hours ☐ 1-2 hours ☐ 30-60min ☐ less often/never

How many hours per day do you as parent(s) watch movies, videoclips, series on TV or on PC / laptop/ tablet on a usual workday?
☐ more than 3 hours ☐ 2-3 hours ☐ 1-2 hours ☐ 30-60min ☐ less often/never

And how many hours on a usual day on the weekend?
☐ more than 3 hours ☐ 2-3 hours ☐ 1-2 hours ☐ 30-60min ☐ less often/never

Does your child watch TV mostly alone or together with you?
☐ mostly alone ☐ often alone ☐ partly alone, partly together ☐ often together ☐ mostly/always together

How often does your child listen to radio plays or audio books?
☐ daily ☐ several times a week ☐ once a week ☐ less often ☐ never

When reading to your child, how do you normally proceed? Please tick the boxes on how often you do the following things:

	Never	Rarely	Sometimes	Often	Always
I ask my child questions about the story	❍	❍	❍	❍	❍
I encourage my child to pronounce words on their own	❍	❍	❍	❍	❍
I ask my child to repeat sentences in the story	❍	❍	❍	❍	❍
I repeat and complete what my child says	❍	❍	❍	❍	❍
I tell stories that go beyond the book's text	❍	❍	❍	❍	❍
I encourage my child to describe the pictures	❍	❍	❍	❍	❍
I relate the stories to our everyday life	❍	❍	❍	❍	❍
We focus on the joy of reading and the time spent together	❍	❍	❍	❍	❍

To what extend do you agree with the following statement: "We like reading in our family"?

☐ very strongly ☐ strongly ☐ slightly ☐ to a lesser extend ☐ not at all

To what extend do you agree with the following statement: "In our family, we often talk about things we've read"?

☐ very strongly ☐ strongly ☐ slightly ☐ to a lesser extend ☐ not at all

To what extend do you agree with the following statement: "My child is very interested in being read to and is looking forward to it"?

☐ very strongly ☐ strongly ☐ slightly ☐ to a lesser extend ☐ not at all

To what extend do you agree: "Reading is an important activity in our family"?

☐ very strongly ☐ strongly ☐ slightly ☐ to a lesser extend ☐ not at all

What does apply to you and your child?	Does not apply at all	Does not apply	Neither wrong nor right	Does apply a little	Does exactly apply
At home, I often explain to my child to read from left to right, what a word or a sentence is and where a sentence starts/ends.	❍	❍	❍	❍	❍
At home, I specifically explain my child letters and how to write them.	❍	❍	❍	❍	❍
At home, I practise the alphabet with my child, e.g. I encourage him/her to recite or sing it.	❍	❍	❍	❍	❍
At home, I encourage my child by using easy words to recognise or read letters and words, e.g. the own name or the name of his/her favourite animal.	❍	❍	❍	❍	❍
At home, we memorise poems, rhymes or songs.	❍	❍	❍	❍	❍

2) Home Numeracy Environment

How often does your child count in everyday life (e.g. when setting the table with tableware or when counting down hours or days until a certain event)?
☐ daily ☐ several times a week ☐ once a week ☐ every 2-3 weeks ☐ less often/never

How often do you play counting games with your child (e.g. “Benjamin Blümchen: Lerne Zählen”, “Die Maus – Lern-Spiel-Sammlung”, “Kosmolino: 1,2,3…”)?
☐ several times a week ☐ once a week ☐ every 2-3 weeks ☐ less often ☐ never

How often do you play arithmetic games with your child (e.g. „Ich lerne Rechnen“, „Zahlen und Rechnen“, „Zählen und Rechnen mit Ernie und Bert“, „1+2=3 Rechnen macht Spaß“)?
☐ several times a week ☐ once a week ☐ every 2-3 weeks ☐ less often ☐ never

How often do you play dice games with your child (e.g. „Mensch ärgere Dich nicht“ or „Tempo, kleine Schnecke“)?
☐ several times a week ☐ once a week ☐ every 2-3 weeks ☐ less often ☐ never

How often do you involve your child in weighing and counting food and paying at the counter when you go shopping?
☐ several times a week ☐ once a week ☐ every 2-3 weeks ☐ less often ☐ never

How often do you involve your child in counting, weighing or measuring ingredients when cooking?
☐ several times a week ☐ once a week ☐ every 2-3 weeks ☐ less often ☐ never

How often do you talk with your child about measurements (e.g. weight, temperature or speed)?
☐ several times a week ☐ once a week ☐ every 2-3 weeks ☐ less often ☐ never

To what extend do the following statements apply to your family?

„In our family, we think that it is important to be able to calculate“?

☐ very strongly ☐ strongly ☐ slightly ☐ to a lesser extend ☐ not at all

„My child shows interest in learning to calculate and to count and is looking forward to it“?

☐ very strongly ☐ strongly ☐ slightly ☐ to a lesser extend ☐ not at all

„Mathematics is important in our family“?

☐ very strongly ☐ strongly ☐ slightly ☐ to a lesser extend ☐ not at all

What does apply to you and your child?	Does not apply at all	Does not apply	Neither wrong nor right	Does apply a little	Does exactly apply
At home, I often explain to my child how to split apples for people or cakes in pieces.	❍	❍	❍	❍	❍
At home, I precisely show my child numbers and how to write them.	❍	❍	❍	❍	❍
At home, I practise simple calculations with my child (e.g. 2+2).	❍	❍	❍	❍	❍
At home, I often talk to my child about the time e.g. by means of the clock or the calendar.	❍	❍	❍	❍	❍
At home, I precisely show my child geometrical forms and patterns and how to draw them.	❍	❍	❍	❍	❍
